# The RNA Silencing Enzyme RNA Polymerase V Is Required for Plant Immunity

**DOI:** 10.1371/journal.pgen.1002434

**Published:** 2011-12-29

**Authors:** Ana López, Vicente Ramírez, Javier García-Andrade, Victor Flors, Pablo Vera

**Affiliations:** 1 Instituto de Biología Molecular y Celular de Plantas (IBMCP), CSIC-UPV, Valencia, Spain; 2 Department of Experimental Sciences, Universidad Jaume I, Castellón, Spain; Indiana University, United States of America

## Abstract

RNA–directed DNA methylation (RdDM) is an epigenetic control mechanism driven by small interfering RNAs (siRNAs) that influence gene function. In plants, little is known of the involvement of the RdDM pathway in regulating traits related to immune responses. In a genetic screen designed to reveal factors regulating immunity in *Arabidopsis thaliana*, we identified *NRPD2* as the *OVEREXPRESSOR OF CATIONIC PEROXIDASE 1 (OCP1)*. *NRPD2* encodes the second largest subunit of the plant-specific RNA Polymerases IV and V (Pol IV and Pol V), which are crucial for the RdDM pathway. The *ocp1* and *nrpd2* mutants showed increases in disease susceptibility when confronted with the necrotrophic fungal pathogens *Botrytis cinerea* and *Plectosphaerella cucumerina*. Studies were extended to other mutants affected in different steps of the RdDM pathway, such as *nrpd1*, *nrpe1*, *ago4*, *drd1*, *rdr2*, and *drm1drm2* mutants. Our results indicate that all the mutants studied, with the exception of *nrpd1*, phenocopy the *nrpd2* mutants; and they suggest that, while Pol V complex is required for plant immunity, Pol IV appears dispensable. Moreover, Pol V defective mutants, but not Pol IV mutants, show enhanced disease resistance towards the bacterial pathogen *Pseudomonas syringae* DC3000. Interestingly, salicylic acid (SA)–mediated defenses effective against *Ps*DC3000 are enhanced in Pol V defective mutants, whereas jasmonic acid (JA)–mediated defenses that protect against fungi are reduced. Chromatin immunoprecipitation analysis revealed that, through differential histone modifications, SA–related defense genes are poised for enhanced activation in Pol V defective mutants and provide clues for understanding the regulation of gene priming during defense. Our results highlight the importance of epigenetic control as an additional layer of complexity in the regulation of plant immunity and point towards multiple components of the RdDM pathway being involved in plant immunity based on genetic evidence, but whether this is a direct or indirect effect on disease-related genes is unclear.

## Author Summary

The influence of epigenetic regulation in controlling the adaptive responses of living organisms to changes in the environment is becoming a common theme in biology. RNA–directed DNA methylation (RdDM) is an epigenetic control mechanism driven by a subset of noncoding small interfering RNAs (siRNAs) that influence gene function without changing DNA sequence by inducing *de novo* methylation of cytosines, or by modification of histones, at their target genomic regions. The implication and roles of the RdDM mechanism in the orchestration of plant immune responses still remains to be characterized. A recent study in the model plant Arabidopsis showed that ARGONAUTE4, one of the characteristic components of the RdDM pathway, was required for plant immunity against bacterial pathogens. Here, in a genetic screen aiming to identify cellular factors integral in regulating immunity in Arabidopsis, we further identified that the RNA polymerases V, another crucial component of the RdDM pathway, is pivotal for plant immunity against fungal pathogens. Similarly, we identified that additional components of the RdDM pathway, but surprisingly not RNA polymerase IV, are similarly required for plant immunity. Based on genetic evidence, our results highlight the importance of RdDM as an additional layer of complexity in the regulation of plant immune responses.

## Introduction

RNA-directed DNA methylation (RdDM) is an epigenetic modification mechanism driven by noncoding small interfering RNAs (siRNAs) [1], [2]. siRNAs are present in most eukaryotic organisms, are highly developed in plants and regulate gene expression at the transcriptional and posttranscriptional level in a sequence-specific manner. In contrast to microRNAs (miRNAs) that are derived from the transcripts of miRNA genes generated by RNA Polymerase II, production of RdDM-associated siRNAs requires RNA Polymerase IV (Pol IV) complex activity which includes, among other constituents, the largest and second largest subunits, NRPD1 and NRPD2, respectively [3]–[5]. Upon the action of Pol IV, the resulting single-stranded RNAs are used as templates for RNA-dependent RNA polymerase 2 (RDR2) generating double-stranded RNAs, which are processed by DICER-LIKE 3 (DCL3) [6], [7]. Subsequently, RNA methyltransferase HUA ENHANCER-1 (HEN1) generates functional siRNAs that are recruited by ARGONAUTE4 (AGO4) to form the AGO4-RISC multiprotein complex guided to siRNA-complementary genome sequences [8]–[10]. AGO4-siRNA complexes interact with the RNA Polymerase V (Pol V) complex, which includes the largest and second largest subunits, NRPE1 and NRPD2, respectively. Pol V is somehow required to recruit DRM2 methyltransferase as well as histone-modifying complexes to finally establish the methylation pattern in the siRNA-complementary genome sequences; however, the details of this recruitment are unknown. This process results in the methylation of certain genome repeat regions and their subsequent transcriptional silencing [2]. Among the different classes of siRNA, the 24 nt in lenght hetrocromatic siRNAs (hc-siRNAs) and repeat-associated siRNAs (ra-siRNAs), primarily derived from transposons, repeated elements and heterochromatin regions, are those functioning in the RdDM pathway by mediating DNA methylation and/or histone modification at the target sites [2].

Small RNAs regulate a multitude of biological processes in plants, including sustaining genome integrity, development, metabolism and responses to changing environmental conditions and abiotic stress [11]. Increasing evidences also indicate that plant endogenous small RNAs, including miRNAs and siRNAs, are integral regulatory components of plant defense machinery against microbial pathogens [12]. The Arabidopsis miR393 imparts basal resistance to the bacterial pathogen *Pseudomonas syringae* DC3000 by targeting the auxin receptors TIR1, ABF2 and ABF3 [13]. Besides miR393, two other miRNA families, miR160 and miR167, are upregulated following *Ps*DC3000 inoculation and target members of auxin-response factors (ARF) [14]. Thus, in response to bacterial infection, plants suppress multiple components of the auxin signaling pathway. In turn, bacteria have developed type III secretion effectors that repress transcription of miRNA genes, the host RNA silencing machinery is suppressed and therefore disease susceptibility increase [15]. Similarly, Lu et al. [16] identified a series of 10 miRNAs families in loblolly pine whose expression were suppressed, and the transcript levels of their target genes increased, upon infection with the rust fungus *Cromartium quercuum* f. sp. *fusiforme*. Likewise, upon infection of *Brasica rapa* with Turnip mosaic virus (TuMV) the miR1885 is upregulated, and its target is predicted to be a member of the TIR-NBS-LRR class of disease-resistance proteins [17]. Thus, it appears that following detection of pathogen-associated molecules, plant cells undergo changes in miRNA global profiles that mediate the establishment of a specific defense response [12], [18].

Although plants contain only several hundred miRNAs, they contain huge numbers of endogenous siRNAs but only in a few cases the involvement of siRNAs in plant immunity has been described. In Arabidopsis, the natural antisense transcript (NAT)-derived nat-siRNAAATGB2 and the long siRNA lsiRNA-1, which specifically targets the mitochondrial pentatricopeptide protein(PPR)-like gene *PPRL* and the RNA-binding protein gene *AtRAP*, respectively, are induced by the bacterial pathogen *Ps*DC3000(*avrRpt2*) and contribute to plant antibacterial immunity [19], [20]. The endogenous siRNAs generated at disease resistance *RPP4* locus, which impart resistance to both the bacterial *Ps* pv. *maculicola* and the oomycetes *Hyaloperonospora arabidopsidis*, constitute a third example for siRNA-mediated resistance responses [21]. However, it remains unclear how RdDM participates in this type of processes.

The understanding of the overall contribution and requirement of the different components that conform the RdDM pathway, and how important they are in the regulation of the RdDM-mediated processes, particularly in relation to plant immunity, is an issue that still remains to be fully understood. Previously we described a genetic screen in Arabidopsis design to identify mutants (*ocp* mutants) with altered immune responses [22]. This allowed identifying AGO4, through the characterization of its mutant allele *ago4-2/ocp11*, as an important component of the RdDM pathway in mediating plant immune responses towards *Ps*DC3000 [23]. Towards characterizing the contribution of other components of the RdDM pathway in plant immunity, we report here on the isolation and characterization of *ocp1*, a recessive mutant allele of *NRPD2*. Our results support that RdDM, through the action of RNA Pol V, is pivotal in modulating immune responses towards pathogens.

## Results/Discussion

### Characterization of *ocp1* Plants

**Figure 1:**
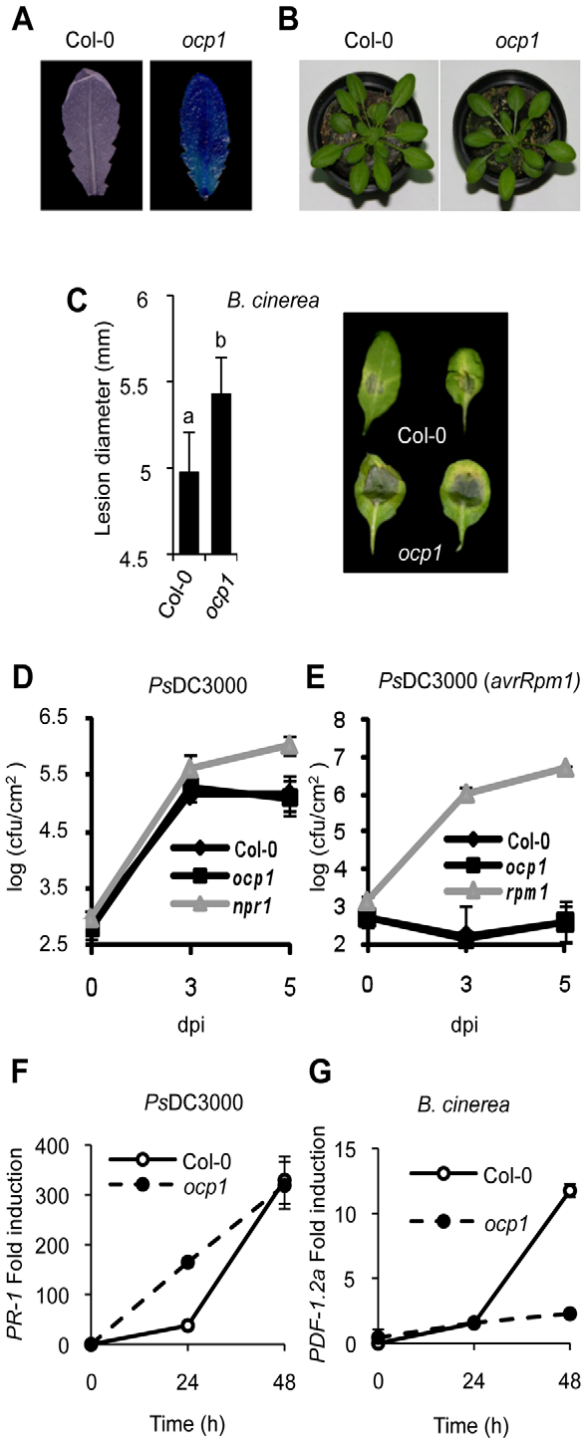
Characterization of *ocp1* plants. (A) Comparative histochemical analysis of GUS activity in rosette leaves from a parental wild-type plant carrying the *P_Ep5C_:GUS* transgene (left), and *ocp1* mutant plant (right). (B) Macroscopic comparison of 3-week-old wild-type (left) and *ocp1* plants (right). (C) Resistance response of wild-type and *ocp1* plants to virulent *B. cinerea*. Lesion size was measured 5 days after inoculation (dpi). Data points represent average lesion size ± SE (n≥30 lesions). Representative leaves from wild-type and *ocp1* plants 4 dpi. (D–E) Growth rates of virulent *Ps*DC3000 (D) and avirulent *Ps*DC3000 (*AvrRpm1*) (E) in Col-0, *ocp1* and *npr1* or *rpm1* plants. (F–G) RT-qPCR expression analysis of *PR-1* (F) and *PDF1.2a* (G) in wild-type and *ocp1* plants at different times following inoculation with *Ps*DC3000 (F) and *B. cinerea* (G). Data represent the mean ± SD; n = 3 biological replicates.

The Arabidopsis *ocp* mutants were identified previously in a genetic screen [22] designed to isolate negative regulators of pathogen-induced defense responses. The H_2_O_2_-responsive and defense-related *Ep5C* gene promoter fused to GUS was used as reporter [24]. Here we described the characterization of the *ocp1* mutant. Figure 1A shows the constitutive *Ep5C::GUS* expression in rosette leaves from *ocp1* plants compared with its parental Col-0 line (line 5.2). *ocp1* plants exhibited similar plant architecture and growth habit to the wild-type plants (Figure 1B). F_1_ hybrids from a backcross between parental and *ocp1* plants showed the absence of GUS activity, and GUS activity segregated in the F_2_ progeny as a single recessive Mendelian locus [*OCP1:ocp1*, 111:33 (P<0.05, χ^2^ test)].

We hypothesize that the constitutive expression of *Ep5C::GUS* observed in *ocp1* plants might be accompanied by an altered disease resistance response to pathogens as previously revealed in *ocp3* and *ocp11* plants [22], [23], [25], [26]. Therefore, we inoculated *ocp1* plants with the virulent necrotrophic fungal pathogen *Botrytis cinerea* and monitored the disease response in leaves in comparison with the parental line. Disease was scored by recording the extent of necrosis. Wild-type plants exhibited normal susceptibility to *B. cinerea* (Figure 1C), with inoculated leaves showing necrosis accompanied by extensive proliferation of the fungal mycelia. In contrast, *ocp1* plants showed increased susceptibility to *B. cinerea* distinguished by moderate but statistical significant enlargement of necrotic areas at inoculation sites (Figure 1C).

Susceptibility of *ocp1* plants to pathogens was also investigated with the bacterial pathogen *Ps*DC3000. The *npr1* mutant, which is compromised in resistance towards this pathogen [27] was used as a control. Resulting bacterial growth in inoculated leaves is shown in Figure 1D and indicates the wild-type and *ocp1* mutant susceptibility was unchanged towards virulent *Ps*DC3000. In addition, plants were inoculated with an avirulent strain of *Ps*DC3000 carrying the *avrRpm1* gene that triggers a hypersensitive cell death response in the plant that stops bacterial growth. The *rpm1* mutant, compromised in the hypersensitive response and consequently hypersusceptible to the pathogen, was used as a control. Results showed the growth of *Ps*DC3000 (*avrRpm1*) in *ocp1* plants was not significantly different to that observed in wild-type plants (Figure 1E). These results were consistent with normal accumulation of transcripts of the salicylic acid (SA)-responsive gene *PR-1* at 48 h following inoculation with *Ps*DC3000 (Figure 1F), however induction occurs earlier in *ocp1* plants. Interestingly, induction of the jasmonic acid (JA)-responsive gene *PDF1.2a*, a characteristic molecular response of plants to fungal attack, was compromised in *ocp1* plants following inoculation with *B. cinerea* (Figure 1G). This later observation is congruent with the observation that *ocp1* plants showed enhanced disease susceptibility to this pathogen (Figure 1C).

### *OCP1* Is At3g23780 and Encodes NRPD2, the Second Largest Subunit of the RNA Pol IV and Pol V

**Figure 2:**
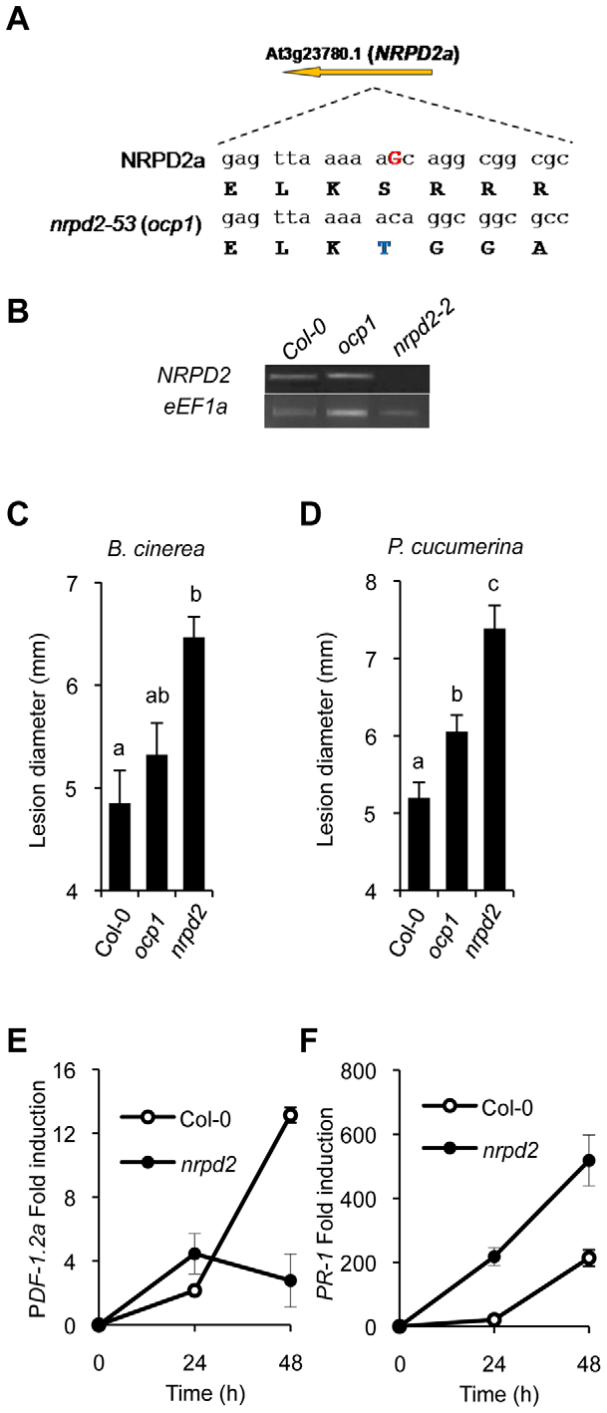
*ocp1* is a mutant allele of *NRPD2*. (A) *OCP1* corresponds to *At2g27040* encoding NRPD2. The G nucleotide residue deleted in the *ocp1* allele is indicated in red bold uppercase letters in the wild-type sequence. Deduced amino acid sequences are indicated below each nucleotide triplet, and the first amino acid change (S to T) where the frameshift of the OCP1 protein starts is shown in blue. (B) *NRPD2* expression level by RT-PCR in mRNAs derived from Col-0, *nrpd2-2* and *ocp1* plants. The *eEF1a* house-keeping gene was used as a control. (C–D) *nrpd2* plants show enhanced susceptibility to fungal pathogens. Lesion size was measured in Col-0, *ocp1* and *nrpd2-2* plants after inoculation with *B. cinerea* (C) or *P. cucumerina* (D). Data points represent average lesion size ± SE (n≥30 lesions). ANOVA detected significant differences at the P<0.05 level. (E–F) RT-qPCR determination of *PDF1.2a* (E) and *PR-1* (F) transcript levels following inoculation with *P. cucumerina*. Data represent the mean ± SD; n = 3 biological replicates.

The genetic lesion carried by *ocp1* plants was identified by positional cloning (Figure S1). A single nucleotide deletion was detected on locus At3g23780, particularly in the third exon of the transcribed gene encoding NRPD2, the second largest subunit of the RNA Pol IV and Pol V protein complexes (Figure 2A and Figure S1C). The loss of a nucleotide residue created a change in the NRPD2 open reading frame that leads to a frame shift starting at residue 595 (Figure 2A) followed by an incorrect 22 amino acid C-terminal tail sequence before an in-frame stop codon (Figure S2). The mutation renders a protein of 616 amino acid residues, instead of the 1172 contained in NRPD2, that thus has lost almost half of the protein sequence, including the amino acids that contribute to the active site of RNA polymerases [28].

The result obtained in our mapping strategy was corroborated with a test of allelism between *ocp1* plants and plants carrying a null allele of *NRPD2*, in particular with *nrpd2-2* plants which carry a T-DNA insertion (SALK_046208) [3]. Analysis of GUS expression driven by the *Ep5C* gene promoter in 20 F1 plants derived from a cross between homozygous *ocp1* plants with homozygous *nrpd2-2* plants or, alternatively, from a reversed cross between *nrpd2-2* plants with *ocp1* plants, revealed that all F1 plants showed constitutive GUS expression (Figure S3). Conversely, control crosses between the parental Col-0 plants carrying the *Ep5C::GUS* gene construct (line 5.2) with either *ocp1* plants or *nprd2-2* plants revealed no GUS expression in any of the F1 22 plants analyzed (Figure S3). The result indicates that the *ocp1* and *nrpd2-2* are mutant alleles of the same *NRPD2* gene and supported the conclusion that the *ocp1* mutation represents a loss of function allele. Hence, the *ocp1* mutation will be referred also as *ocp1*/*nrpd2-53*.

From the type of mutation found, we cannot exclude the possibility that *ocp1* plants are still able to produce a truncated version of the NRPD2 protein with a residual ability to interact with other components of the RNA polymerase complexes. Since Pol IV and Pol V complexes are comprised of a variety of interacting subunits, some being polymerase-specific while other subunits shared (i.e., NRPD2) [5], [29], [30], and with some cross-talk described for some of their subunits (i.e., between NRPD2 and NRPE1; [4]), we can not discard the possibility that the relationships between the different components of the two RNA polymerase complexes may become differentially altered in the *ocp1* mutant. In this respect, the availability of the *ocp1* allele may represent a valuable experimental tool to approach the biochemical regulation of the RdDM mechanism.

Interestingly, RT-PCR analyses of *NRPD2* transcript levels in *ocp1* plants revealed the absence of notable changes in gene expression compared with Col-0 plants (Figure 2B). This is in marked contrast with the expression observed in *nrpd2-2* null mutant plants where no transcript amplification products can be obtained (Figure 2B). A comparison of the disease resistance response between *ocp1* and *nrpd2-2* plants revealed that while the *ocp1* plants showed a moderate increase in susceptibility to *B. cinerea*, the *nrpd2-2* null mutant responded to *B. cinerea* infection with a remarkable enhancement in susceptibility (Figure 2C). The enhanced susceptibility phenotype of *nrpd2-2* plants was further corroborated by recording the susceptibility towards *Plectosphaerella cucumerina*, a different fungal necrotroph (Figure 2D). Consistent with the observed increase in disease susceptibility to *P. cucumerina*, RT-qPCR experiments revealed that induction of the JA-responsive *PDF1.2a* gene was disabled in *nrpd2-2* plants compared to Col-0 (Figure 2E). These results mirror what occurs in *ocp1* plants following *B. cinerea* infection (Figure 1F). Of importance for understanding the immune-related phenotype of *nrpd2-2* plants is the observation that expression of the SA-responsive *PR-1* gene was clearly enhanced following fungal inoculation in the mutant when compared to wild-type plants (Figure 2F). Since *nrpd2-2* plants show an enhanced disease susceptibility of bigger magnitude than that observed in *ocp1*/*nrpd2-53* plants, subsequently, the experiments related to disease resistance/susceptibility will be carried out employing the *nrpd2-2* allele.

### *SUPERMAN*, *5S* Genes, and the *AtSN1* Retroelement Are Hypomethylated in *ocp1* Plants

**Figure 3:**
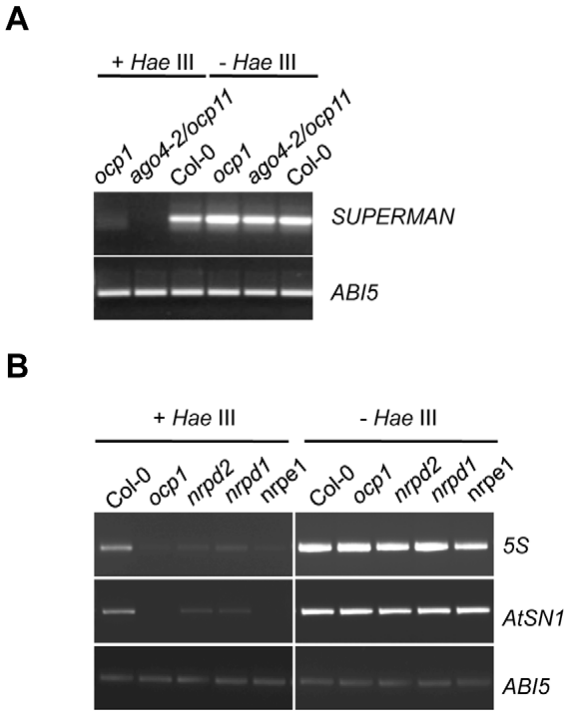
*ocp1* plants show hypomethylation in RdDM target DNA sequences. Genomic DNA isolated from Col-0, *ocp1* and *ago4-2/ocp11* plants (A) and *nrpd2*, *nrpd1* and *nrpe1* (B) was digested (+) or not (−) with *Hae*III and amplified by PCR for *SUPERMAN* promoter (A), the ribosomal *5S* genes and the retrotransposon *AtSN1* (B). *ABI5* contains no target sequences for *Hae*III and was used as a control.

To further substantiate the molecular phenotype of *ocp1* plants in relation to RdDM, we checked if the methylation status of different RdDM target sequences could be similarly affected in *ocp1* and *nrpd2-2* plants. We analyzed the methylation status in *ocp1* plants of the RdDM pathway DNA target sequences *SUPERMAN*, ribosomal *5S* genes and the retrotransposon *AtSN1* [31]. We used methylation tests employing the methylation-sensitive restriction endonuclease *Hae*III (where *Hae*III will not cut DNA if methylated), with subsequent amplification by PCR [32]. Initial experiments revealed that *ocp1*, as well as *ago4-2/ocp11* plants used as controls, exhibit a higher degree of hypomethylation in *SUPERMAN* gene compared to Col-0 plants (Figure 3A). Analyses were extended to the ribosomal *5S* genes and the *AtSN1* retrotransposon and we incorporated *nrpd2-2*, *nrpd1-3* and *nrpe1-1* mutants for comparison. Figure 3B shows mutants demonstrated higher degrees of hypomethylation in the sequences analyzed. DNA samples derived from *ocp1* plants exhibited decreased amplification for the *5S* and *AtNS1* loci, confirming a clear DNA methylation deficiency in this mutant. The *ABI5* gene, whose sequence contains no restriction sites for *Hae*III, was used as a control. Methylation tests were also used to ascertain whether or not the enhanced induction observed for the *PR-1* gene, or the repression of *PDF1.2a*, in the *nrpd2* mutant following fungal infection correlated with defects in the DNA methylation of their promoter regions. Since both genes contain a large number of recognition sites for the methylation-sensitive restriction enzymes *FspE*I, *MspJ*I and *Ava*II (168 target sequences in the *PR-1* gene and 298 targets in the *PDF1.2a* gene), and where *FspE*I and *MspJ*I sites must be methylated for the enzymes to cleave the DNA, we used restriction analysis with these enzymes with subsequent amplification by PCR to check the methylation status of the *PR-1* and *PDF1.2a* genes. The results shown in Figure S4 and Figure S5 revealed that none of the promoters appear methylated, not even in Col-0 plants. Conversely, the sensitivity of the methylated *5S* ribosomal DNA (Figure S4) to the aforementioned enzymes revealed the appropriateness of the method used to identify methylation of cytosine residues. The lack of a methylation footprint in the DNA of the defense-related *PR-1* and *PDF1.2a* genes might suggest that the abnormal expression patterns concurring in *nrpd2* mutant plants must obey not to a direct modification of cytosine residues but to other type of chromatin modification or mechanism similarly controlled either directly or indirectly by the RdDM pathway.

### The Pol V Complex, But Not Pol IV, Is Required for the Correct Immune Response against *B. cinerea* and *P. cucumerina*

**Figure 4:**
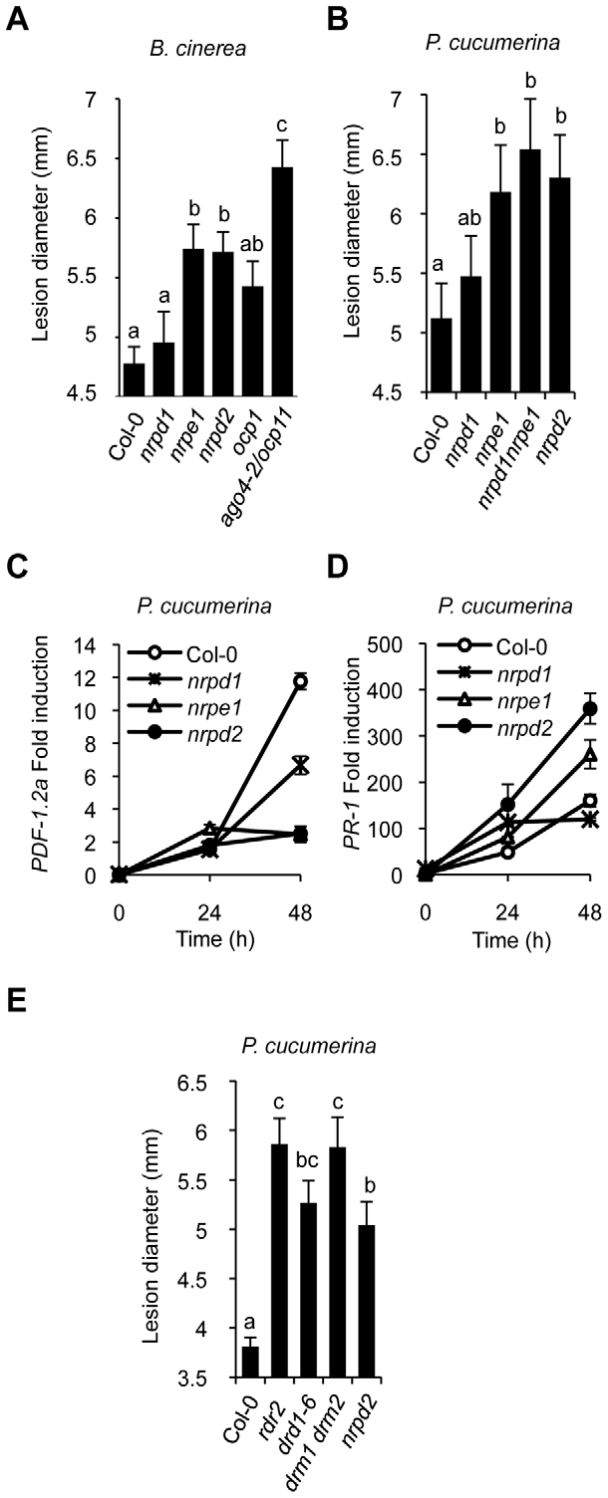
Comparative immune responses of RdDM mutants to inoculation with *B. cinerea* and *P. cucumerina*. (A) Disease susceptibility of Col-0, *nrpd1*, *nrpe1*, *nrpd2*, *ocp1* and *ago4-2/ocp11* plants to *B. cinerea*. (B) Comparative disease susceptibility of the Pol IV and Pol V defective mutants to *P. cucumerina*. (C–D) RT-qPCR of *PDF1.2a* (C) and *PR-1* (D) transcript levels following inoculation with *P. cucumerina in* Col-0, *nrpd1, nrpe1 and nrpd2* plants. Data represent the mean ± SD; n = 3 biological replicates. (E) Comparative disease susceptibility of *rdr2*, *drd1*, *drm1drm2* and *nrpd2* mutants to *P. cucumerina*. ANOVA detected significant differences at the P<0.05 level.

As for NRPD2, we addressed if other RdDM pathway components are similarly engaged in plant immunity. A comparative analysis of the disease resistance response of *nrpd1*, *nrpe1*, and *ago4* mutant plants due to inoculation by *B. cinerea* was performed in relationship to *nrpd2*. Figure 4A shows an increase in *nrpe1* disease susceptibility to *B. cinerea*; the susceptibility being of a magnitude similar to that attained in *nrpd2* plants. This enhancement in susceptibility was comparatively greater than that observed in *ocp1* plants but less than in *ago4-2/ocp11* plants. Conversely, *nrpd1* plants did not exhibit a significant deviation from the normal disease response observed in Col-0 plants. This differential behavior was further corroborated in the Pol IV and Pol V defective mutants by challenging with *P. cucumerina* (Figure 4B). The *nrpd1 nrpe1* double mutant that would be defective in both Pol IV and Pol V activities was incorporated in this experiment for comparison. *nrpd1 nrpe1* plants showed an enhanced disease susceptibility of a magnitude similar to that attained in *nrpe1* or *nrpd2* plants. Furthermore, fungal biomass determination in leaves inoculated with *P. cucumerina*, as an alternative method for recording disease resistance, also revealed that the single *nrpd2* and *nrpe1* mutants, as well as the double *nrpd1 nrpe1* mutant support significantly more fungal growth than Col-0 and the *nrpd1* mutant (Figure S6). Therefore, the Pol V complex participates in the regulation of the immune response to necrotrophs while the Pol IV complex appears at least partially dispensable. This is sustained also by the observation that expression patterns of the *PDF1.2a* and the *PR-1* genes in *nrpd1* plants are dissimilar to that commonly attained in both *nrpe1* and *nrpd2* plants, either in the course of infection with *P. cucumerina* (Figure 4C–4D) or upon chemical induction by treating plants with a solution of either 0.5 mM SA (Figure S7A) or 0.1 mM JA (Figure S7B). Notorious is the higher JA-triggered *PDF1.2* gene induction in *nrpd1* plants in comparison to Col-0 (Figure S7B). Conversely, this JA-triggered *PDF1.2a* gene induction is notably repressed in the Pol V defective mutants (Figure 4C and Figure S7B). This is in marked contrast with the altered expression pattern observed in *nrpd2* plants where induction of *PR-1* gene expression showed enhancement following inoculation with *P. cucumerina* (Figure 4D) or upon external application of SA (Figure S7A). Importantly, this pattern of gene expression was reproduced in *nrpe1* plants (Figure 4D and Figure S7A). Moreover, the transcription factors WRKY6 and WRKY53 that bind W-box and transcriptionally regulate gene expression of SA-related genes, including *PR-1* [33], and are themselves induced by pathogen infection [34], show similar enhanced level of induction following SA application in *nrpd2* and *nrpe1* plants when compared to Col-0 or *nrpd1* plants (Figure S7C and S7D).

### *drd1*, *rdr2*, and *drm1drm2* Mutants Show Compromised Immune Responses to *P. cucumerina*

To further extent the implication of RdDM mechanism in plant immunity we inoculated the *drm1drm2* double mutant plants, which is compromised in *de novo* DNA methylation [35], with *P. cucumerina* and recorded the disease response. Results in Figure 4E reveal that loss of the functional RDM methyltransferase compromise disease resistance and results in plants showing enhanced susceptibility to *P. cucumerina* to levels even higher than in *nrpd2* plants. This reinforces the proposal that RdDM is pivotal for plant immunity. Likewise, we observed that the chromatin-remodeling factor DRD1, which is required for the association of NRPE1 with chromatin [36], is also pivotal for plant immunity. Figure 4E shows that *drd1-6* plants phenocopy Pol V defective mutants. The RNA-dependent RNA polymerase 2 (RDR2), which functions early in the RdDM pathway by generating dsRNA from the ssRNA transcripts thought to emanate presumably from the the Pol IV complex was also entertained in these experiments. Intriguingly, *rdr2* plants also show a strikingly enhancement in susceptibility to fungal infection (Figure 4E), achieving highest levels of susceptibility to *P. cucumerina*. This observation strongly argues in favor of RDR2 as required for plant immunity. Then, how can a mechanism explain the exclusion of RNA Pol IV in mediating plant immunity while the rest of downstream components of the RdDM pathway are engaged in this biological process? There are previous evidences where Pol V has been described to operate independently of Pol IV, such as in the mechanism for maintaining the methylation status of target sequences [37], and thus for some processes Pol IV and Pol V act not in concert [38]. A hypothesis that could help explain the paradoxical observations indicating that Pol V, RDR2, AGO4, DRD1 and DRM2, but not Pol IV, are required for plant immunity to fungal pathogens could be one where RDR2 can accept RNA transcripts derived from the action of RNA Pol V, and not necessarily only from RNA Pol IV. These putative transcripts thought to be acted upon by RDR2, which generates dsRNA, will be processed into siRNAs and feed into the RdDM pathway. In support for the existence of Pol V-dependent transcripts required for DNA methylation and silencing is the recent identification of low-abundance intergenic non-coding (IGN) transcripts [36]. It could be that a similar situation is on the basis to explain the requirement of RdDM for plant immune responses. This possibility merits future research approaches.

### SA–Mediated Defense Genes Are Poised for Enhanced Activation by Histone Modifications in Pol V Defective Mutants

The previous results suggest that in Pol V defective mutants SA-related defense genes are poised for enhanced activation following perception of pathogenic cues and concurrently JA-related defenses appear impeded for induction. This will be congruent with a notion where Pol V may regulate a priming phenomenon for SA-mediated defense responses that ultimately would modulate the speed and extent of gene activation. However, the lack of a methylation footprint in the DNA of the defense-related *PR-1* and *PDF1.2a* genes (Figure S5 and Figure S6) suggest that the observed abnormal gene expression patterns concurring in the Pol V defective mutants is not to be due to an altered DNA methylation pattern resulting from a defective RdDM pathway. However, one could still entertained the possibility that changes in chromatin structure such as those obeying to covalent modification of histones, which are also under the control of the RdDM pathway, may be on the basis for the enhanced expression observed for *PR-1* and, therefore, for the altered resistance phenotypes in the mutant plants. This would be congruent with the recent identification of a mechanism linking chromatin modification in wild type plants, through the differential modification of histones in several genes encoding WRKY transcription factors (i.e. WRKY6, WRKY29 or WRKY53), with priming of a defense response following pharmacological treatment with the SA analogue acidobenzolar S-methyl (BTH) which functions as a priming agent in plants [39]. Thus, we hypothesized that in Pol V defective mutants *PR-1* could be poised for enhanced activation of gene expression by a differential modification of histones.

**Figure 5:**
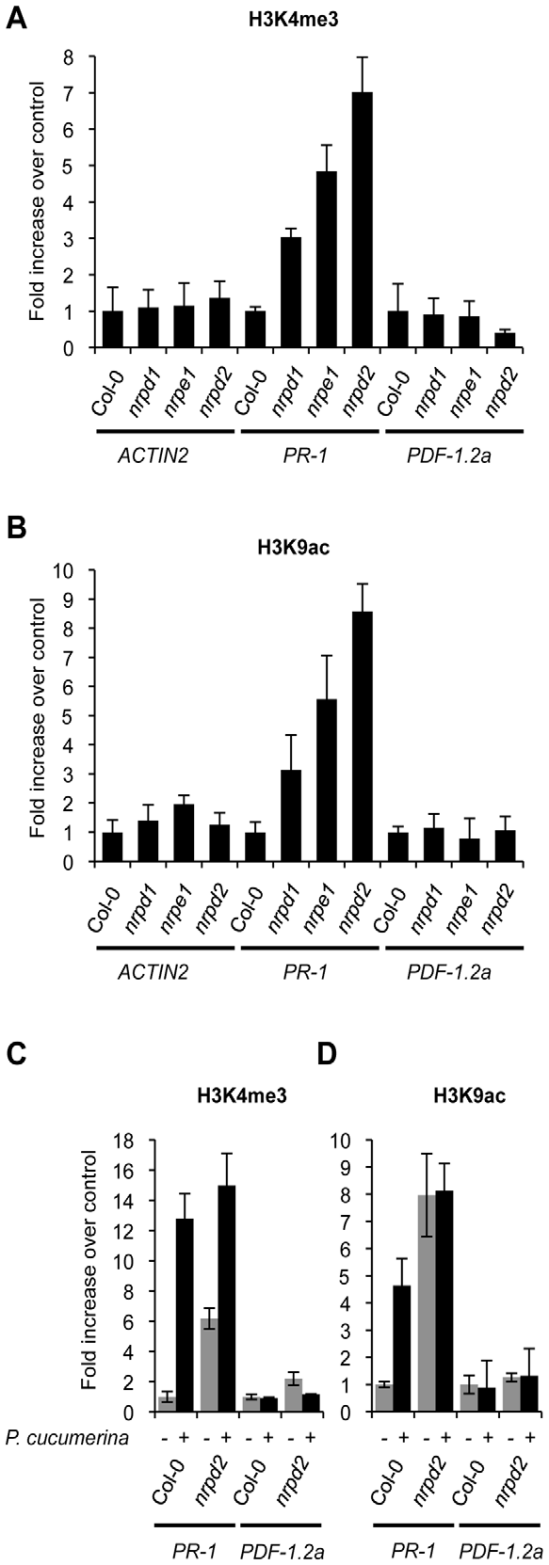
Histone H3 modifications. Comparative level of histone modifications of *PR-1*, *PDF1.2a* and *Actin2* gene promoters as present in leaf samples from Col-0, *nrpd1*, *nrpe1* and *nrpd2* plants. (A) Histone H3 Lys4 trimethylation (H3K4me3) on the indicated gene promoters. (B) Histone H3 K9 acetylation (H3K9ac) on the indicated gene promoters. Data are standardized for Col-0 histone modification levels. (C–D) H3K4me3 (C) and H3K9ac (D) modifications on *PR-1* and *PDF1.2a* gene promoters in Col-0 and *nrpd2* plants 48 h after inoculation with *P. cucumerina*. (D) (−) mock inoculated plants, (+) *P. cucumerin* inoculated plants. Data are standardized for mock inoculated Col-0 histone modification levels. Data represent the mean ± SD; n = 3 biological replicates.

By using chromatin immunoprecipitation (ChIP) we analyzed trimethylation of histone H3 Lys4 (H3K4me3) and acetylation of histone H3 Lys9 (H3K9ac) on the promoter of the *PR-1* gene. For comparison, the promoter of the JA-inducible *PDF1.2a* gene, that of the constitutively expressed *Actin2* gene and also those of the *WRKY6* and *WRKY53* genes were similarly studied. The specificity of the ChIP reaction was evaluated in advance by measuring histone modifications on these genes in Col-0 plants treated with BTH (Figure S8A and S8B). On the *PR-1* promoter H3K4me3 and H3K9ac marks increased after BTH application while these marks did not change in the promoters of *Actin2* or *PDF1.2a* (Figure S8A and S8B). As for *PR-1*, these chromatin marks were similarly increased in the promoters of *WRKY6* and *WRKY53* upon treatment with BTH (Figure S8C). Thus chromatin marks normally associated with active genes [39], [40] are set in the promoters of SA-related defense genes by the priming stimulus of BTH. Interestingly, determination of H3K4me3 (Figure 5A) and H3K9ac (Figure 5B) chromatin marks in the *PR-1* promoter in ChIP samples derived from *nrpd2* and *nrpe1* plants, revealed that these marks are already set in these two mutants, although *PR-1* gene activation does not take place. Thus, Pol V defective mutants mimic Col-0 plants treated with the priming agent BTH. This reconciles with the idea that the *PR-1* gene is switch on for priming in the Pol V defective mutant and explains why this gene shows enhanced induction upon pathogenic attack in the same mutants (Figure 4D). In the *nrpd1* mutant only a moderate increase in the setting of these chromatin marks in the promoter of *PR-1* was detected (Figure 5A and 5B). No variation in similar activation marks was observed in the promoters of the *Actin2* and *PDF1.2a* genes (Figure 5A and 5B). Other histone marks, such as H3K9me2 and H3K27me3, both of which repressive marks normally associated with heterochromatin and established through the RdDM pathway [41], appear notably reduced in the *PR-1* promoter in ChIP samples derived from *nrpd2* and *nrpe1* plants, and much less reduced in *nrpd1* plants, when compared to Col-0 plants (Figure S9A and S9B). Moreover, Col-0 plants respond to *P.cucumerina* infection with reduction in the setting of these two repressive histone marks in the *PR-1* gene promoter but not in the promoters of the *PDF1.2a* or *Actin2* genes (Figure S9C). The dismantling of histone repressive marks in infected plants, along with the concurring increase in histone activation marks and decrease in repressive marks in the promoter of the *PR-1* gene, as observed in *nrpd2* and *nrpe1* plants, gives further support to the implication of Pol V in regulating defense gene activation.

As for *PR-1*, H3K4me3 activation marks are also constitutively set in the promoters of the *WRKY6* and *WRKY53* genes in healthy *nrpd2* and *nrpe1* plants (Figure S8C), again mirroring the effect carried out by BTH on Col-0 for these promoters (Figure S8C). Further analysis demonstrated that Col-0 plants respond to *P. cucumerina* infection with a drastic increase in the setting of H3K4me3 and H3K9ac activation marks in the promoters of *PR-1* (Figure 5C and 5D). In *nprd2* plants, in which these chromatin marks are already set in *PR-1*, *P. cucumerina* inoculation further increase H3K4me3 marks on the *PR-1* promoter to levels that are even higher than those attained in Col-0 (Figure 5C). However, for H3K9ac marks no further increase was observed in *nrpd2* plants, suggesting that this type of mark is completely set in the mutant. In contrast, no variation in the setting of these chromatin marks was detected in the *PDF1.2a* promoter upon fungal infection (Figure 5C and 5D). For *WRKY6* and *WRKY53* gene promoters, Col-0 plants respond to *P. cucumerina* infection by similarly increasing H3K4me3 mark setting in both promoters (Figure S10). Compared to Col-0, *nrpd2* plants constitutively carry increased H3K4me3 mark setting in *WRKY6* and *WRKY53* gene promoters and do not show further increases upon inoculation, but instead slightly decrease (Figure S9). Together, these data imply that Pol V, either directly or indirectly, regulates the extent of chromatin modifications on SA defense-related gene promoters, and may be the underlying mechanism controlling priming marks facilitating the more rapid activation of gene expression observed upon perception of pathogenic cues. As reported for other genes, the observed covalent modifications in chromatin might provoke increases in the accessibility of DNA or perhaps in the provision of docking sites for gene activators [42], [43].

### *nrpd2* and *nrpe1* Plants Show Enhanced Resistance to *Ps*DC3000

**Figure 6:**
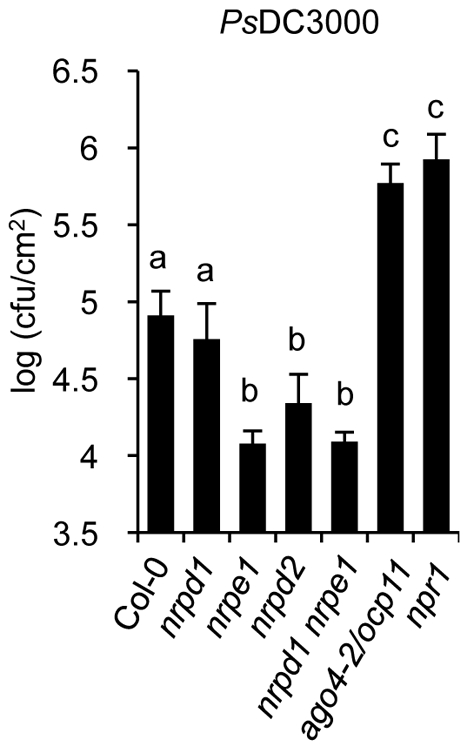
Comparative immune responses of Pol IV and Pol V defective mutants to inoculation with *Pseudomonas syringae* DC3000. Growth rates of *Ps*DC3000 in Col-0, *nrpd1*, *nrpe1*, *nrpd2*, and *nrpd1 nrpe1* plants. The *Ps*DC3000 disease susceptible mutants *ago4-2/ocp11* and *npr1* were included for comparison. Data represent the mean ± SD; n = 3 biological replicates.

Enhanced activation of SA-mediated defenses is characteristic of plants resistant to biotrophic pathogens, like *Ps*DC3000, and is on the basis for a systemic type of immunity known as systemic acquired resistance (SAR) [44]. Our results on a priming effect for enhanced expression of SA defense-related genes in *nrpd2* and *nrpe1* plants suggest these mutants may be altered in the resistance to *Ps*DC3000. Consequently, we addressed Pol IV and Pol V defective mutants in search for defects in the immune response to *Ps*DC3000. We used *ago4-2*/*ocp11* and *npr1* plants as controls, both exhibiting heightened *Ps*DC3000 disease susceptibility [23], [27]. Interestingly, a significant enhanced disease resistance to *Ps*DC3000 was observed in *nrpd2*, *nrpe1*, and in *nrpd1 nrpe1* plants, when compared to Col-0 plants (Figure 6). In contrast, statistically significant effects were not observed in *nrpd1* plants relative to Col-0 in response to *Ps*DC3000, giving further support to the idea that RNA Pol IV seems not engaged in plant immunity. The observed heightened resistance towards *Ps*DC3000 in *nrpd2* and *nrpe1* plants indicated that in wild-type plants Pol V is required for susceptibility to this pathogen. However, in *ago4-2/ocp11* plants resistance to *Ps*DC3000 is severely compromised. Although there is no obvious explanation for this contrasting effect, as previously stated [23] one can speculate that AGO4 can serve a novel function, and while required for an effective defense response it may operate independently of the RdDM pathway.

An important observation derived from the results presented is the co-existence of an enhanced disease resistance to a biotrophic bacteria, like *Ps*DC3000, with an enhanced susceptibility to necrotrophic fungi in Pol V defective mutants. This reveals an underlying complexity in the control of disease resistance by RdDM. The SA and JA signal pathways are under an antagonistic equilibrium that occasionally culminates with the partial inhibition of one pathway when the other is facilitated. Consequently the interaction between pathways serves to optimize responses to a specific type of pathogenic insult [45]. Our results demonstrated that *nrpd2* and *nrpe1* plants are poised for enhanced activation of SA defense-related genes and respond to pathogen attack with a marked enhancement in the induced expression of marker genes, which suggests these plants are more prone to mobilize the defense arsenal controlled by SA. A simpler explanation for these observations is that in wild type plants Pol V negatively regulates a priming mechanism for SA-mediated disease resistance while keeping intact a JA-mediated disease resistance. Defects in Pol V function, such as those observed in *nrpd2* and *nrpe1* mutants, de-repress the priming mechanism for SA-mediated resistance through pertinent chromatin modifications, and renders enhanced resistance to *Ps*DC3000. As a tradeoff, presumably mediated through endogenous antagonistic cross talk mechanisms, mis-regulation of the JA-mediated disease resistance occurs. This thus explaining the repressed expression of JA-marker gene and the heightened susceptibility of *nrpd2* and *nrpe1* plants to fungal pathogens. However, although this mechanism seems very likely, we still cannot disregard the possibility that RdDM may be similarly required for normal expression of one or more unknown genes involved in JA signaling. Disruption of RdDM could thus lead to a disruption of JA signaling which would in turn result in hyper-activation of SA signaling. In fact, mutant plants with JA-mediated signaling pathway defects and hypersensitivity to fungal necrotrophs concurrently present a less repressed SA-mediated signaling pathway, resulting in a more efficient defense response when challenged with biotrophic pathogens [45], [46]. Experiments directed towards identification of an epigenetic footprint associated to the JA pathway merits future reach and will help clarify the complexity of the antagonistic cross-talk mechanism between the SA and the JA signal transduction pathways.

A deeper understanding on how the RdDM and associated chromatin modification acts as a mechanism controlling gene priming and induced immune responses in plants, and how pathogens may counteract this epigenetic regulation for their own benefit will open new avenues for the a better knowledge on how plant immunity is orchestrated.

## Materials and Methods

### Plant Material and Growth Conditions

*Arabidopsis* were grown in a growth chamber (19 to 23°C, 85% relative humidity, 100 µE m^−2^ s^−1^ fluorescent illumination) under a 10/14 h light/dark photoperiod. All mutants are in Col-0 background. *ago4-2/ocp11*, *npr1*, *rpm1-1*, *rdr2*, *drd1-6* and *drm1/drm2* plants were previously described [23]. *nrpd2-2* (SALK_046208); *nrpe1-11* (SALK_029919) and *nrpd1-3* (SALK_128428) were obtained from the Salk Institute Genomic Analysis Laboratory (http://signal.salk.edu/). *nrpd1 nrpe1* double mutant was obtained from T. Lagrange.

### GUS Staining

Plant leaves were incubated overnight at 37°C in GUS staining buffer as previously described [22].

The *ocp1* mutant was backcrossed twice to the *P_Ep5C_:GUS* line to confirm its recessive inheritance. *ocp1* plants were crossed to L*er*, and F_1_ plants were allowed to self. F_2_ plants were scored for co-segregation of high constitutive GUS activity with simple sequence length polymorphisms (SSLP) [40]. Molecular markers were derived from the polymorphism database between the L*er* and Col-0 ecotypes (http://www.arabidopsis.org).

### PCR-Based Methylation Assays

Methylation tests using the methylation-sensitive endonuclease *Hae*III, *FspE*I, *Ava*II and *MspJ*I were performed as described [32]. The relative DNA fragment amounts corresponding to *SUPERMAN*, *5S* and *AtSN1* were obtained after 30, 25 and 35 respective PCR cycles. For *ABI5*, 30 (A) or 26 (B) PCR cycles were used. *PR-1* and *PDF1.2a* methylation assays are provided in a supplemental file.

### Expression Analysis

Gene expression analysis, by either RT-PCR or RT-qPCR was performed as described previously [23]. The primers used to amplify the different genes and DNA regions, and the PCR conditions employed for genotyping T-DNA insertions, and RT-PC and qRT-PC experiments are provided in the supporting information file Text S1.

### Bacterial and Fungal Bioassays

Bacterial strains were grown overnight and used to infect 5-week-old *Arabidopsis* leaves by infiltration and bacterial growth determined as described [23]. Twelve samples were used for each data point and represented as the mean ± SEM of log c.f.u./cm^2^. *B. cinerea* and *P. cucumerina* bioassays were performed as previously described [24]. Fungal disease symptoms were evaluated by determining the lesion diameter (in mm) of a minimum of 30 lesions. All experiments were repeated at least three times with similar results.

### Chromatin Immunoprecipitation

Chromatin isolation and immunoprecipitation were performed as described [47]. Chip samples, derived from three biological replicates, were amplified in triplicate and measured by quantitative PCR using primers for *PR-1*, *WRKY6*, *WRKY53* and *Actin2* as reported [39]. The rest of primers are described in Text S1. All ChIP experiments were performed in three independent biological replicates. The antibodies used for immunoprecipitation of modified histones from 2 g of leaf material were antiH3K4m3 (#07-473 Millipore), antiH3K4ac (#07-352 Millipore), antiH3K9me2 (ab1772 Abcam) and antiH3K27me3 (ab6002 Abcam).

## Supporting Information

Figure S1 *ocp1* is *At3g23780* and Encodes NRPD2. To identify the genetic lesion carried by *ocp1* plants, we performed positional cloning of the mutation. To map the position of *ocp1* in the genome, we crossed *ocp1* plants to Landsberg *erecta* (L*er*) plants, and F_2_ plants were scored for co-segregation of high constitutive GUS activity with simple sequence length polymorphisms (SSLP) [48]. An initial analysis of 40 *ocp1* individuals allocated the *ocp1* mutation in chromosome III, between markers nga162 and AthGAPAB which define an interval of 22.2 cM. Further analysis of 472 plants with 12 new polymorphic markers allowed narrowing the position of *ocp1* to an interval of 246.291 pb located between markers CER455355 and CER454777 and comprising 6 BAC clones (A). Four new SSLP markers and one dCAPF (*Derived Cleaved Amplified Polymorphic Sequences*) marker were analyzed for this mapping interval, and we deduced that the *ocp1* lesion was located between markers CER457821 and CER457824, delimiting an interval of 36 kb that comprised a region of 10 ORFs (B). DNA sequencing of this 36 kb interval allowed us to find a guanosine residue deleted in the third exon of the *NRPD2* gene (C).
(TIF)

Figure S2 Comparative amino acid sequences of NRPD2 and OCP1. In blue is indicated the 22 extra amino acid residues preceding the premature stop codon arising due to the nucleotide deletion identified in the *ocp1* mutant. In red is indicated the S to T transition due to the change in the open reading frame as a consequence of the deleted nucleotide.
(TIF)

Figure S3 *ocp1* is allelic to *nrpd2*. The result obtained in our cloning strategy was corroborated with a test of allelism between *ocp1* plants and plants carrying the *nrpd2-2* allele. Analysis of GUS expression driven by the *Ep5C* gene promoter in 20 F1 plants derived from a cross between homozygous *ocp1* plants with homozygous *nrpd2-2* plants or, alternatively, from a reversed cross between *nrpd2-2* plants with *ocp1* plants, revealed that all F1 plants showed constitutive GUS expression. Conversely, control crosses between the parental Col-0 plants carrying the *Ep5C::GUS* gene construct (line 5.2) with either *ocp1* plants or *nprd2-2* plants revealed no GUS expression in any of the 22 F1 plants analyzed. These complementation analyses indicate that the *ocp1* and *nrpd2* are mutant alleles of the same *NRPD2* gene. Hence, the *ocp1* mutation will be referred also as *ocp1/nrpd2-53*.
(TIF)

Figure S4 *PR-1* and *PDF1.2*a genes appear not to be methylated in their DNA sequences. Genomic DNA isolated from Col-0, *nrpd2*, *nrpd1* and *nrpe1* plants were digested (+) or not (−) with *FspE*I, *MspJ*I or *Ava*II and amplified by PCR using specific primers for the indicated promoter regions. The ribosomal *5S* DNA sequences, which are methylated, were used as a control.
(TIF)

Figure S5 Nucleotide sequence of *PR-1* and *PDF1.2a* 5′ promoter regions. Restriction sites for *FspE*I (green) and *MspJ*I (blue) endonucleases are indicated by color sequences. Red circle marks *Ava*II restriction site. Arrows denote position of primers used to amplify the respective promoter regions as indicated in supplemental Methods. The ATG translation initiation codon for the transcribed genes is shown in bold.
(TIF)

Figure S6 Growth of *P. cucumerina* on leaves from Col-0, *nrpd1*, *nrpe1*, *nrpd1 nrpe1* and *nrpd2* plants quantified by qPCR. Plants were inoculated with *P. cucumerina* by spraying full expanded leaves with a solution containing 5×10^6^ spores/ml. Five days after inoculation DNA was extracted from leaves and the amount of the *P. cucumerina β-tubulin* gene quantified by qPCR. Data are standarized for the presence of the *P. cucumerina β-tubulin* gene in Col-0. Data represent the mean ± SD; n = 3 biological replicates.
(TIF)

Figure S7 Transcript abundance by RT-qPCR on control genes following spray treatment with SA and JA. Abundance of *PR-1* (A), *WRKY6* (C) and *WRKY53* (D) transcripts in Col-0, *nrpd1*, *nrpe1* and *nrpd2* plants 48 h after spraying with a solution containing (+) or not containing (−) 0.5 mM SA. (B) Abundance of *PDF1.2a* transcripts in Col-0, *nrpd1*, *nrpe1* and *nrpd2* plants 48 h after spraying with a solution containing (+) or not containing (−) 0.1 mM JA. Data represent the mean ± SD; n = 3 biological replicates.
(TIF)

Figure S8 Histone modifications on control genes and effect of the priming agent BTH. (A–B) Histone H3K4me3 (A) and H3K9ac (B) modifications on *Actin2*, *PR-1* and *PDF1.2a* gene promoters after treatment of Col-0 plants for priming with 0.1 mM BTH (+) or a wettable powder (−) as a control. (C) Comparative level of histone H3K4me3 modification on *WRKY6* and *WRKY53* gene promoters in Col-0, *nrpe1* and *nrpd2* plants and after treatment for gene priming of Col-0 plants with 0.1 mM BTH. Data are standardized for Col-0 histone modification levels. Data represent the mean ± SD; n = 3 biological replicates.
(TIF)

Figure S9 Histone H3K9me2 and H3K27me3 modifications in *PR-1*, *PDF1.2a* and *Actin2* genes in Col-0, *nrpd1*, *nrpe1* and *nrpd2* plants. Comparative levels of histone H2K9m2 (A) and H3K27me3 (B) modifications on *Actin2*, *PR-1* and *PDF1.2a* gene promoters in Col-0, *nrpd1*, *nrpe1* and *nrpd2* plants. (C) Comparative levels of H2K9m2 and H3K27me3 modifications in *Actin2*, *PR-1* and *PDF1.2a* gene promoters in Col-0 plants before and after inoculation with *P. cucumerina*. Data are standardized for non-treated Col-0 histone modification levels. Data represent the mean ± SD; n = 3 biological replicates.
(TIF)

Figure S10 Histone H3K4me3 modification on WRKY6 and WRKY53 gene promoters in Col-0 and *nrpd2* plants following inoculation with *P. cucumerina*. Comparative levels of induced modifications in histone H3K4me3 marks on the promoters of *WRKY6* and *WRKY53* following inoculation of Col-0 and *nrpd2* plants with *P. cucumerina*. BTH-induced H3K4me3 modifications in Col-0 plants are included for comparison of the magnitude of the induced modifications in the two genes. Data are standardized for non-treated Col-0 histone modification levels. Data represent the mean ± SD; n = 3 biological replicates.
(TIF)

Text S1 Primer sequences.
(DOCX)
