# Supplementary figures and images for: The RNA Silencing Enzyme RNA Polymerase V Is Required for Plant Immunity
Source: PLoS Genet. 2011 Dec 29;7(12):e1002434. doi: 10.1371/journal.pgen.1002434 (PMC3248562; doi:10.1371/journal.pgen.1002434)

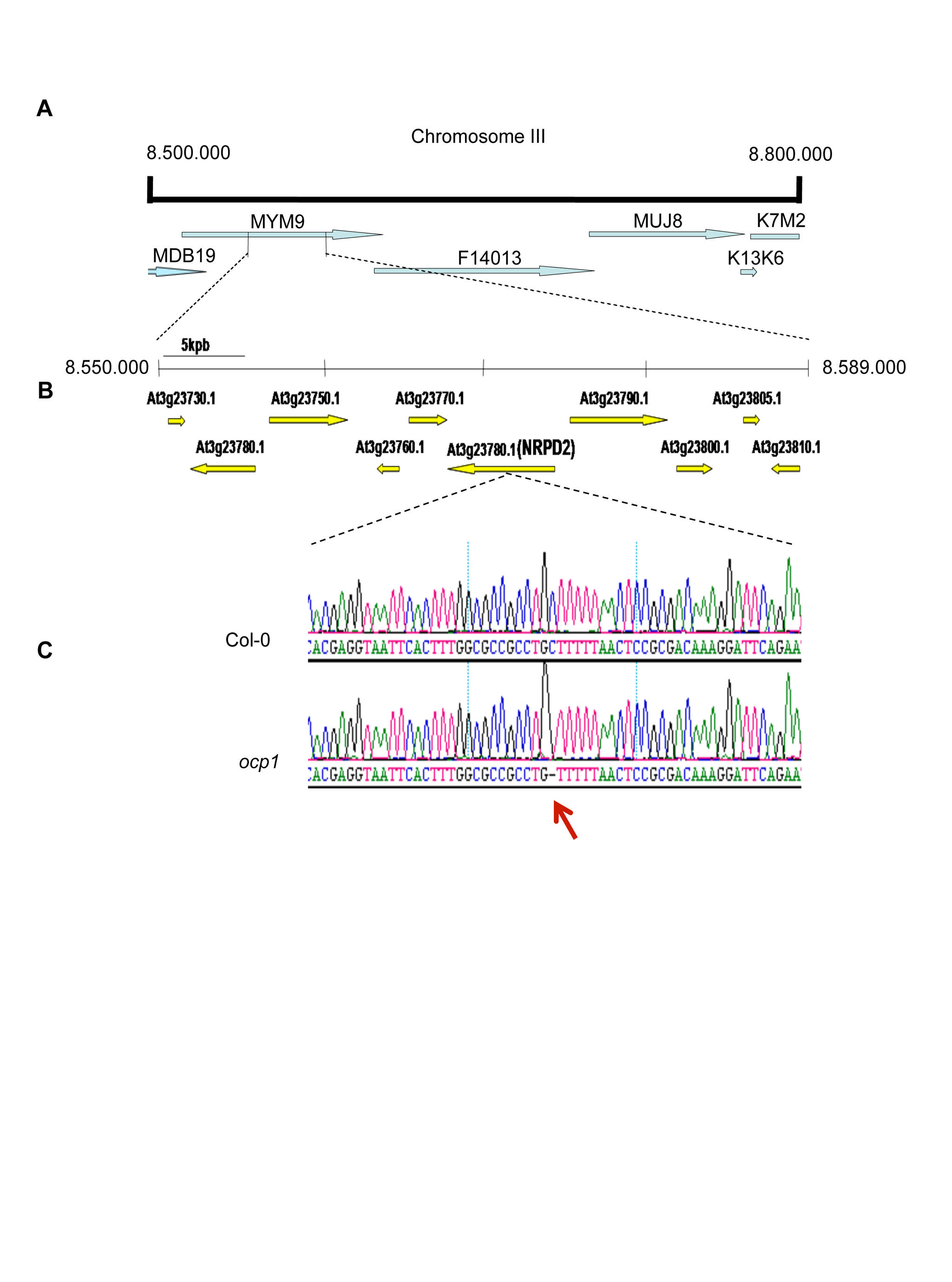

Supplement: Figure S1 — ocp1 is At3g23780 and Encodes NRPD2. To identify the genetic lesion carried by ocp1 plants, we performed positional cloning of the mutation. To map the position of ocp1 in the genome, we crossed ocp1 plants to Landsberg erecta (Ler) plants, and F2 plants were scored for co-segregation of high constitutive GUS activity with simple sequence length polymorphisms (SSLP) [48]. An initial analysis of 40 ocp1 individuals allocated the ocp1 mutation in chromosome III, between markers nga162 and AthGAPAB which define an interval of 22.2 cM. Further analysis of 472 plants with 12 new polymorphic markers allowed narrowing the position of ocp1 to an interval of 246.291 pb located between markers CER455355 and CER454777 and comprising 6 BAC clones (A). Four new SSLP markers and one dCAPF (Derived Cleaved Amplified Polymorphic Sequences) marker were analyzed for this mapping interval, and we deduced that the ocp1 lesion was located between markers CER457821 and CER457824, delimiting an interval of 36 kb that comprised a region of 10 ORFs (B). DNA sequencing of this 36 kb interval allowed us to find a guanosine residue deleted in the third exon of the NRPD2 gene (C). (TIF) [file pgen.1002434.s001.tif]

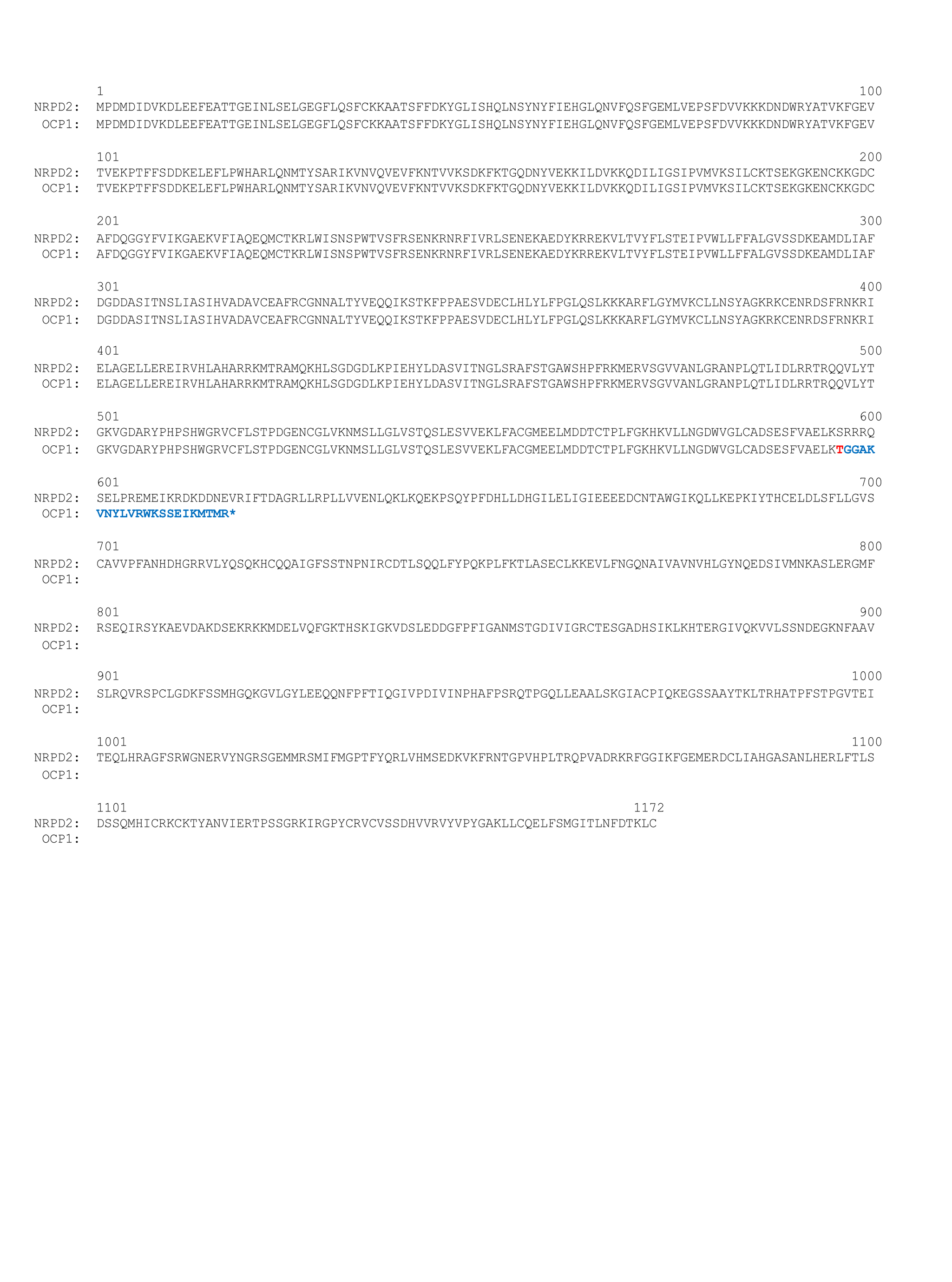

Supplement: Figure S2 — Comparative amino acid sequences of NRPD2 and OCP1. In blue is indicated the 22 extra amino acid residues preceding the premature stop codon arising due to the nucleotide deletion identified in the ocp1 mutant. In red is indicated the S to T transition due to the change in the open reading frame as a consequence of the deleted nucleotide. (TIF) [file pgen.1002434.s002.tif]

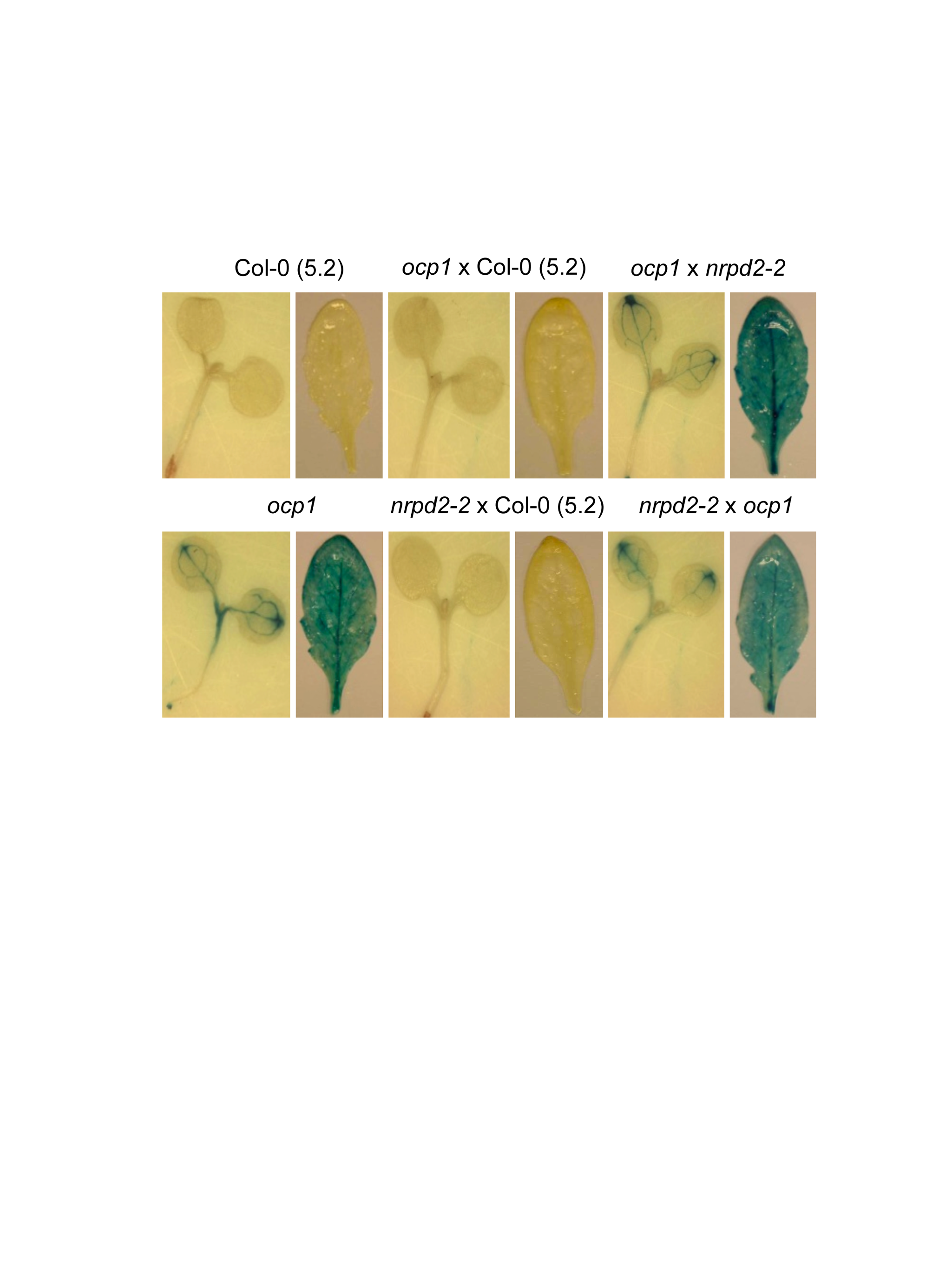

Supplement: Figure S3 — ocp1 is allelic to nrpd2. The result obtained in our cloning strategy was corroborated with a test of allelism between ocp1 plants and plants carrying the nrpd2-2 allele. Analysis of GUS expression driven by the Ep5C gene promoter in 20 F1 plants derived from a cross between homozygous ocp1 plants with homozygous nrpd2-2 plants or, alternatively, from a reversed cross between nrpd2-2 plants with ocp1 plants, revealed that all F1 plants showed constitutive GUS expression. Conversely, control crosses between the parental Col-0 plants carrying the Ep5C::GUS gene construct (line 5.2) with either ocp1 plants or nprd2-2 plants revealed no GUS expression in any of the 22 F1 plants analyzed. These complementation analyses indicate that the ocp1 and nrpd2 are mutant alleles of the same NRPD2 gene. Hence, the ocp1 mutation will be referred also as ocp1/nrpd2-53. (TIF) [file pgen.1002434.s003.tif]

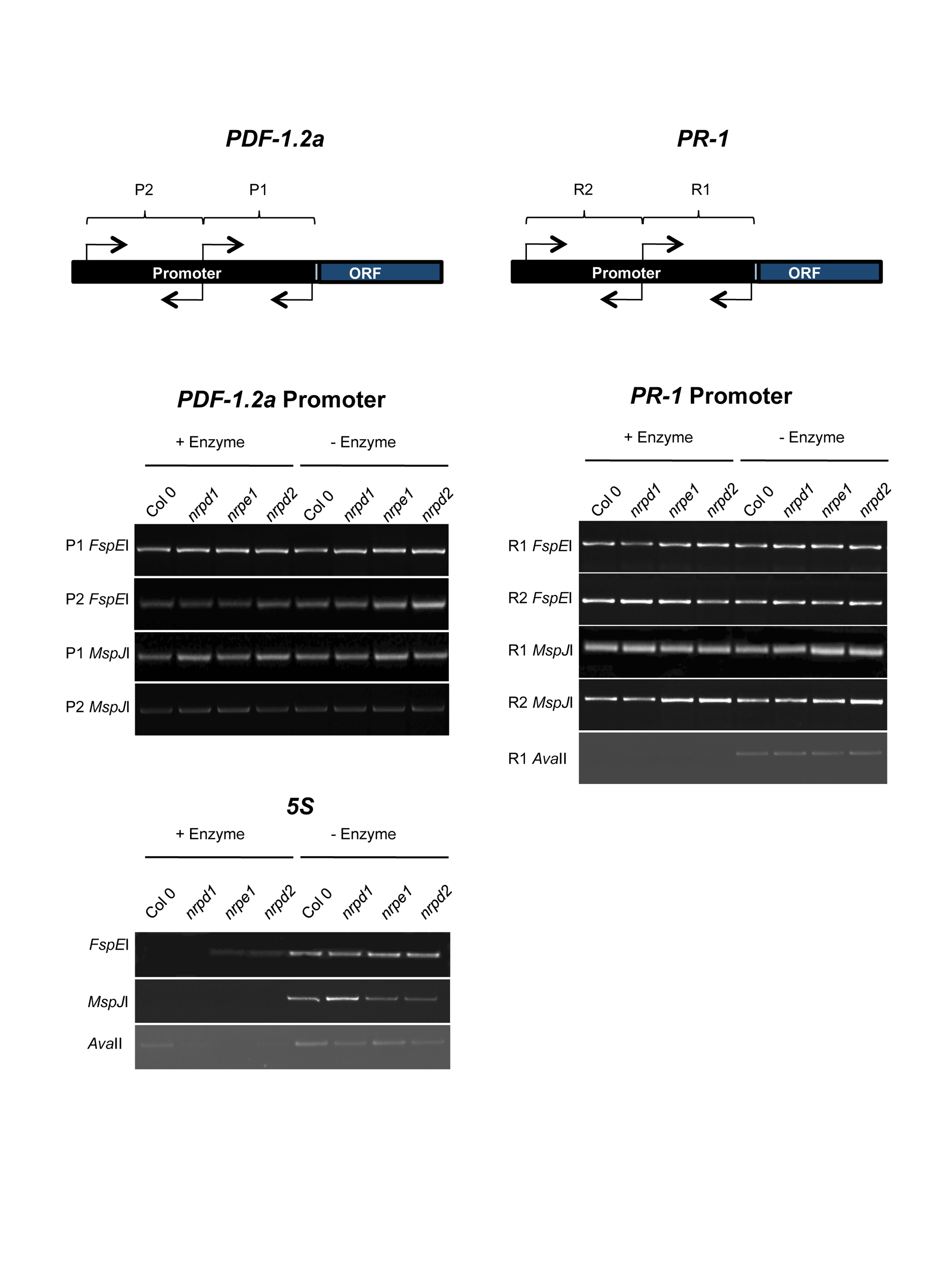

Supplement: Figure S4 — PR-1 and PDF1.2a genes appear not to be methylated in their DNA sequences. Genomic DNA isolated from Col-0, nrpd2, nrpd1 and nrpe1 plants were digested (+) or not (−) with FspEI, MspJI or AvaII and amplified by PCR using specific primers for the indicated promoter regions. The ribosomal 5S DNA sequences, which are methylated, were used as a control. (TIF) [file pgen.1002434.s004.tif]

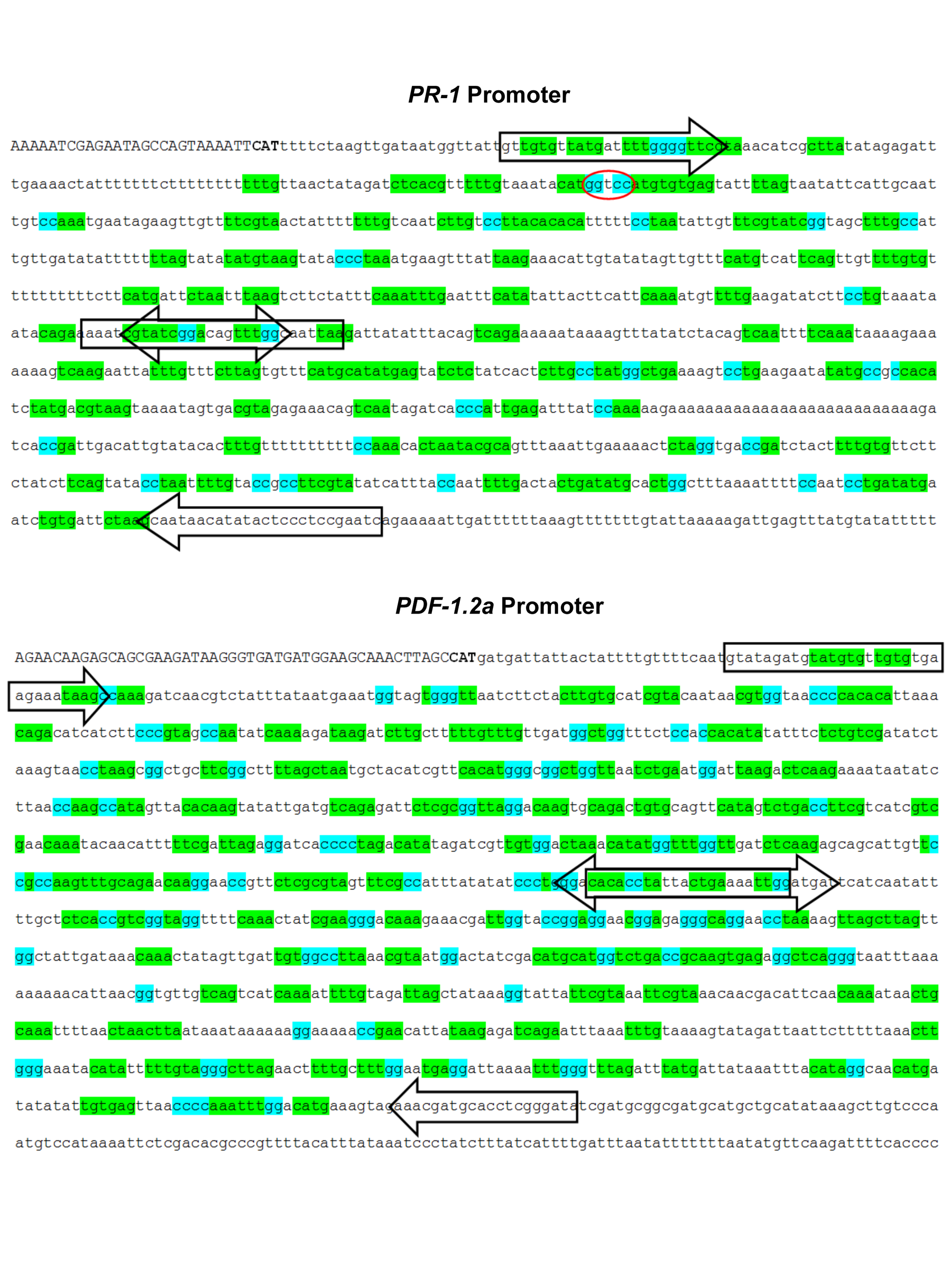

Supplement: Figure S5 — Nucleotide sequence of PR-1 and PDF1.2a 5′ promoter regions. Restriction sites for FspEI (green) and MspJI (blue) endonucleases are indicated by color sequences. Red circle marks AvaII restriction site. Arrows denote position of primers used to amplify the respective promoter regions as indicated in supplemental Methods. The ATG translation initiation codon for the transcribed genes is shown in bold. (TIF) [file pgen.1002434.s005.tif]

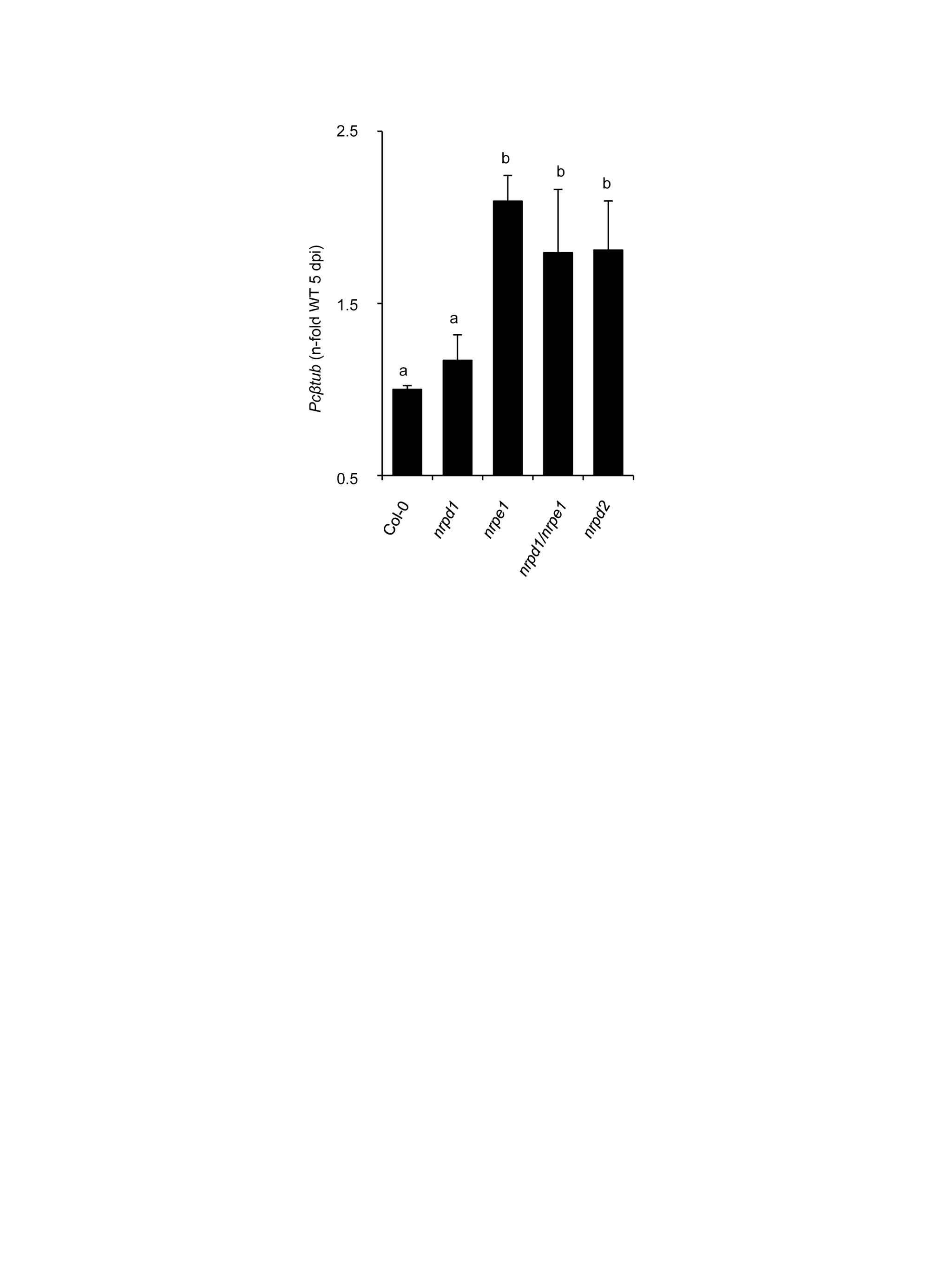

Supplement: Figure S6 — Growth of P. cucumerina on leaves from Col-0, nrpd1, nrpe1, nrpd1 nrpe1 and nrpd2 plants quantified by qPCR. Plants were inoculated with P. cucumerina by spraying full expanded leaves with a solution containing 5×10^6^ spores/ml. Five days after inoculation DNA was extracted from leaves and the amount of the P. cucumerina β-tubulin gene quantified by qPCR. Data are standarized for the presence of the P. cucumerina β-tubulin gene in Col-0. Data represent the mean ± SD; n = 3 biological replicates. (TIF) [file pgen.1002434.s006.tif]

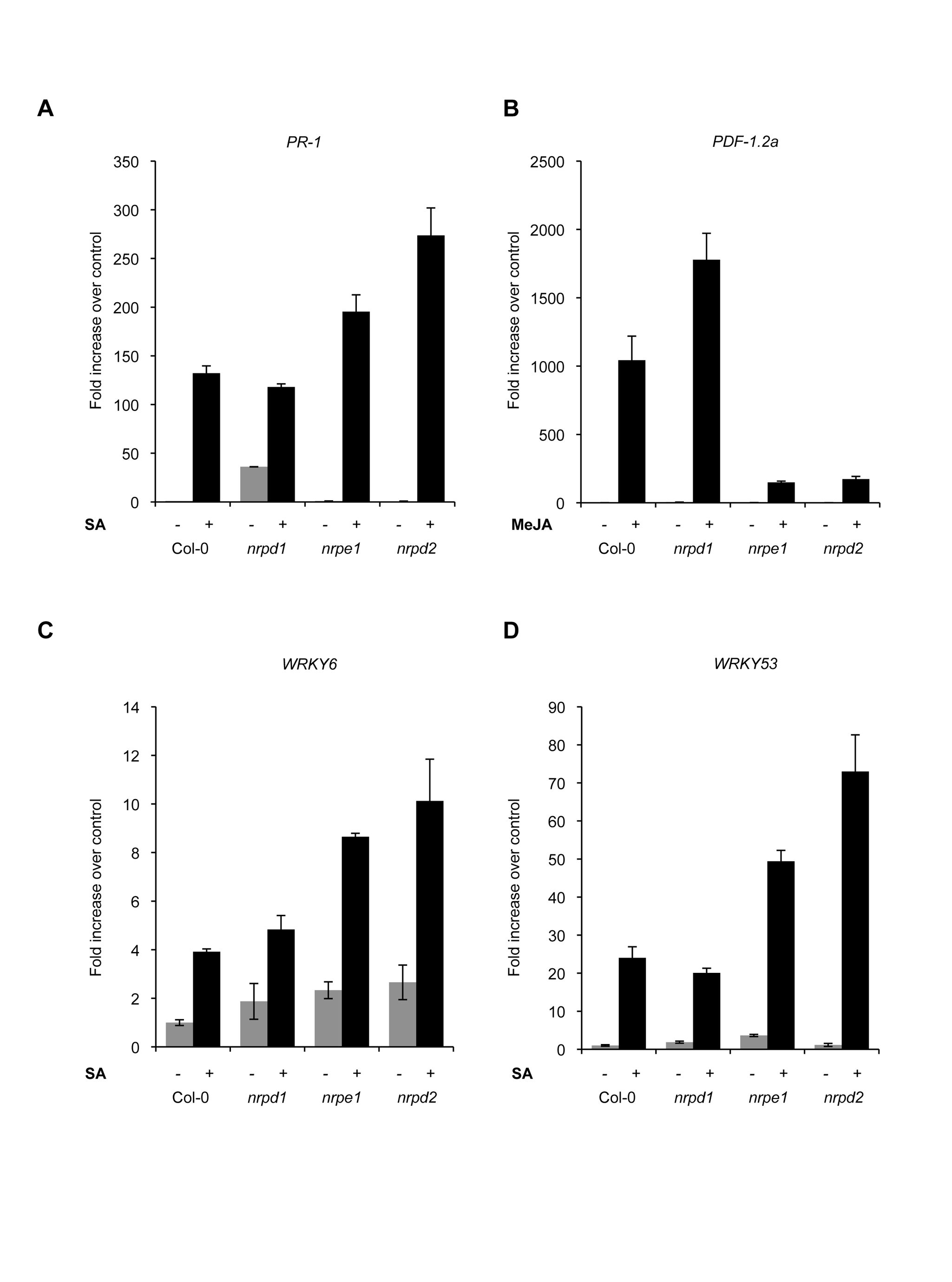

Supplement: Figure S7 — Transcript abundance by RT-qPCR on control genes following spray treatment with SA and JA. Abundance of PR-1 (A), WRKY6 (C) and WRKY53 (D) transcripts in Col-0, nrpd1, nrpe1 and nrpd2 plants 48 h after spraying with a solution containing (+) or not containing (−) 0.5 mM SA. (B) Abundance of PDF1.2a transcripts in Col-0, nrpd1, nrpe1 and nrpd2 plants 48 h after spraying with a solution containing (+) or not containing (−) 0.1 mM JA. Data represent the mean ± SD; n = 3 biological replicates. (TIF) [file pgen.1002434.s007.tif]

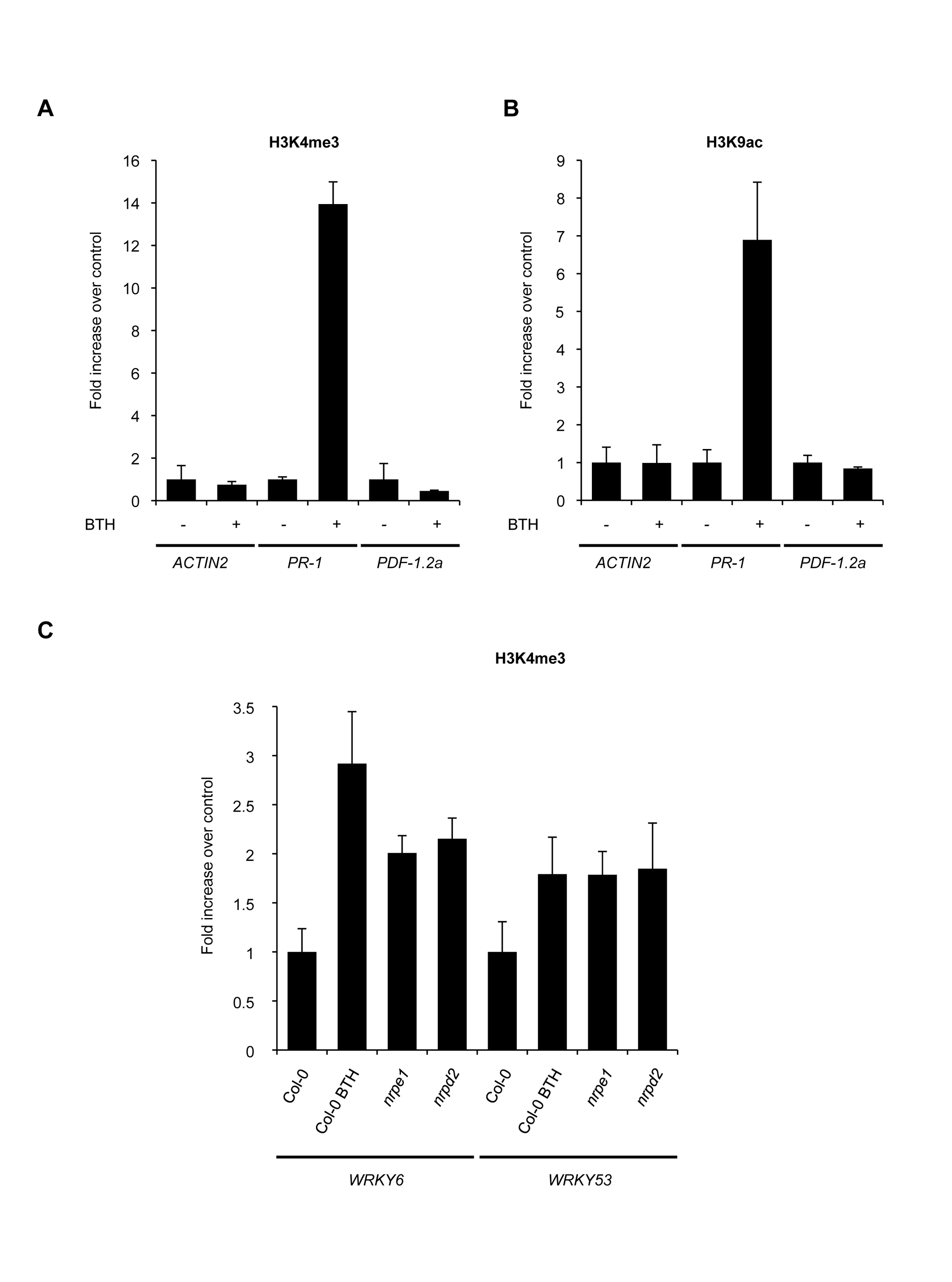

Supplement: Figure S8 — Histone modifications on control genes and effect of the priming agent BTH. (A–B) Histone H3K4me3 (A) and H3K9ac (B) modifications on Actin2, PR-1 and PDF1.2a gene promoters after treatment of Col-0 plants for priming with 0.1 mM BTH (+) or a wettable powder (−) as a control. (C) Comparative level of histone H3K4me3 modification on WRKY6 and WRKY53 gene promoters in Col-0, nrpe1 and nrpd2 plants and after treatment for gene priming of Col-0 plants with 0.1 mM BTH. Data are standardized for Col-0 histone modification levels. Data represent the mean ± SD; n = 3 biological replicates. (TIF) [file pgen.1002434.s008.tif]

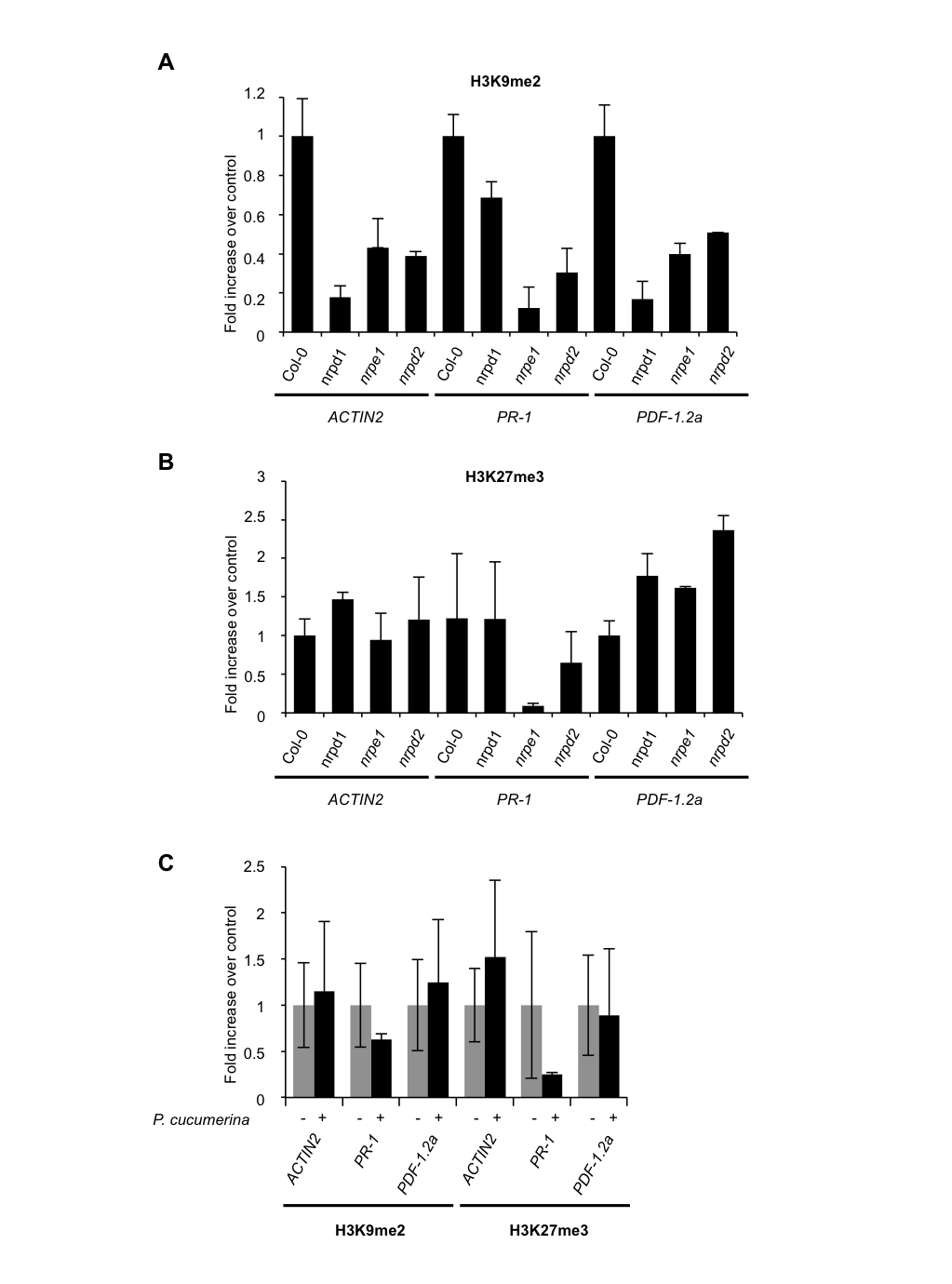

Supplement: Figure S9 — Histone H3K9me2 and H3K27me3 modifications in PR-1, PDF1.2a and Actin2 genes in Col-0, nrpd1, nrpe1 and nrpd2 plants. Comparative levels of histone H2K9m2 (A) and H3K27me3 (B) modifications on Actin2, PR-1 and PDF1.2a gene promoters in Col-0, nrpd1, nrpe1 and nrpd2 plants. (C) Comparative levels of H2K9m2 and H3K27me3 modifications in Actin2, PR-1 and PDF1.2a gene promoters in Col-0 plants before and after inoculation with P. cucumerina. Data are standardized for non-treated Col-0 histone modification levels. Data represent the mean ± SD; n = 3 biological replicates. (TIF) [file pgen.1002434.s009.tif]

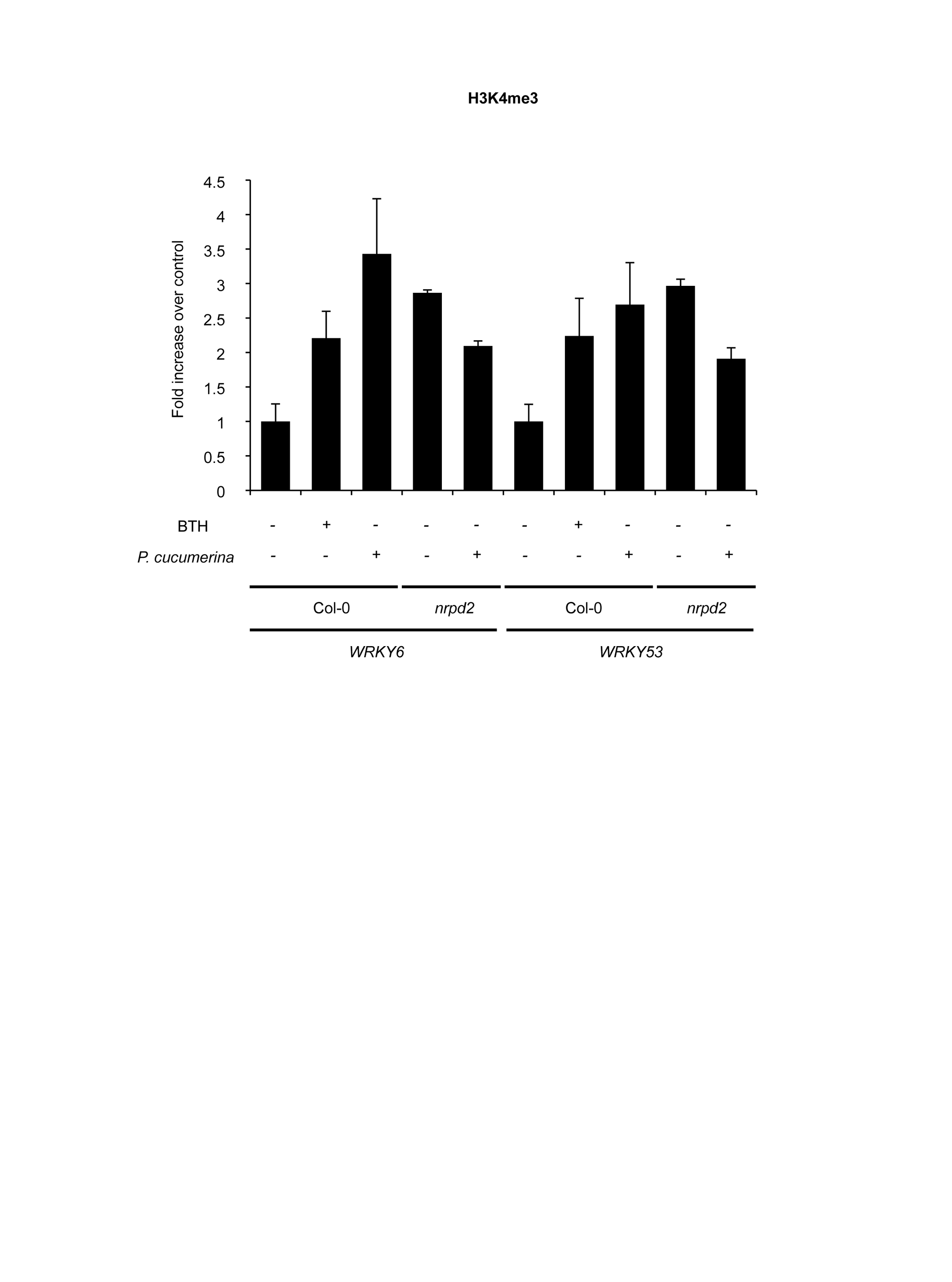

Supplement: Figure S10 — Histone H3K4me3 modification on WRKY6 and WRKY53 gene promoters in Col-0 and nrpd2 plants following inoculation with P. cucumerina. Comparative levels of induced modifications in histone H3K4me3 marks on the promoters of WRKY6 and WRKY53 following inoculation of Col-0 and nrpd2 plants with P. cucumerina. BTH-induced H3K4me3 modifications in Col-0 plants are included for comparison of the magnitude of the induced modifications in the two genes. Data are standardized for non-treated Col-0 histone modification levels. Data represent the mean ± SD; n = 3 biological replicates. (TIF) [file pgen.1002434.s010.tif]
