# Supplementary material for: The RNA Silencing Enzyme RNA Polymerase V Is Required for Plant Immunity
Source: PLoS Genet. 2011 Dec 29;7(12):e1002434. doi: 10.1371/journal.pgen.1002434 (PMC3248562; doi:10.1371/journal.pgen.1002434)
Supplement: Text S1 — Primer sequences. (DOCX) [file pgen.1002434.s011.docx]

**Text S1. Primer sequences.**

**Genotyping T-DNA insertion lines**:

Left border primer: SALK LBb1.3: 5´-ATTTTGCCGATTTCGGAAC-3´

| **GENE** | **Allele** | **PRIMER** | **Sequence (5´---------- 3´)** | **T-DNA Insertion** |
| --- | --- | --- | --- | --- |
| *NRPD1* AT1G63020 | SALK_128428 | direct | CTCAAGTGACCCGTATTCGAACC | Direct+LBb1.3 |
|  | *nrpd1-3* | reverse | GGACCAGCCAATCAGCCTTC |  |
| *NRPD2* AT3G23780 | SALK_046208 | direct | CCGTAAGTGAATTCCACCTCCTC | LBb1.3+Reverse |
|  | *nrpd2-2* | reverse | CCAAGGGAGAAAGAAACAAGAAAGA |  |
| *NRPE1* AT2G40030 | SALK_029919 | direct | CATCATTCTCCTAGCAAACAACATG | LBb1.3+Reverse |
|  | *nrpe1-11* | reverse | GACATTTCTCAGCCGCTTTCTC |  |

**RT-PCR expression analyses:**

| **GENE** | **PRIMERS SET (5´------3´)** | **Nº CYCLES** |
| --- | --- | --- |
| *PR1*  AT2G1414610 | ATGAATTTTACTGGCTATTC | 26 |
|  | AACCCACATGTTCACGGCGGA |  |
| *PR2* AT3G57260 | GCTTCCTTCTTCAACCCCACA | 28 |
|  | CTGAACCTTCCTTGAGACGGA |  |
| *PDF1.2a*  AT5G4442 | ATGGCTAAGTTTGCTTCCAT | 36 |
|  | ACATGGGACGTAACAGATAC |  |
| *NRPD2* AT3G23780 | GATGCTAGATATCCGCACCCC | 33 |
|  | CAGCTCTTCCATTCCACAAGC |  |
| *eEF1a* AT5G60390 | GCACAAGTCATTGATGCCCCA | 22 |
|  | CCTCAAGAAGAGTTGGTCCCT |  |
| *UBC21* AT5G25760 | ATGCAGGCATCAAGAGCGCGACTGT | 26 |
|  | GAATTGAACCCTCTCACATCACCAG |  |

**qRT-PCR expression:**

| **GENE** | **PRIMERS SET (5´------ 3´)** |
| --- | --- |
| *PR1*  AT2G1414610 | ACACGTGCAATGGAGTTTGTGG |
|  | TTGGCACATCCGAGTCTCACTG |
| *PDF1.2a*  AT5G4442 | CTTGTTCTCTTTGCTGCTTTCGAC |
|  | TTGGCTCCTTCAAGGTTAATGCAC |
| *UBC21* AT5G25760 | TGCGACTCAGGGAATCTTCTA |
|  | CATCATCCTTTCTTAGGCATAGC |

**qRT-PCR ChIP**:

*PDF1.2a*

AT5G4442

FW: 5´-AGAGATTCTCGCGGTTAGGA-3

RV: 5´-GCTGCTCTTGAGATCAACCA-3

**Fungal DNA** (*P. cucumerina b-tubulin* gene)

Fw : 5-CAAGTATGTTCCCCGAGCCGT-3

Rv : 5-GAAGAGCTGACCGAAGGGACC-3

**Methylation test:**

| **TARGET** | **PRIMERS SET (5´----------- 3´)** | **Nº CYCLES** |
| --- | --- | --- |
| *5s* | CTCCGCAGTTAAGCGACCTC | 25 |
|  | CTACTCTCGCCCAAGCACGCT |  |
| *AtSN1* | GTTGTATAAGTTTAGTTTTAATTTTACTGGATCTAGTATTAATTT | 35 |
|  | CAATATACAGATCCAAAAAACAAGTTATTAAAATAATATCTTAA |  |
| *ABI5* | TTCATTCCCGGGTCAAGCTAT | 30 |
|  | CCCTTAGCCCTCCCATATCTACT |  |
| R1 *(PR1)* | GTTGTGTTATGATTTTGGGGTTCG | 40 |
|  | CTTAATTGCCAAACTGTCCGATACG |  |
| R2 *(PR1)* | AAATCGTATCGGACAGTTTGGC | 40 |
|  | GATTCGGAGGGAGTATATGTTATTGC |  |
| P1 *(PDF-1.2a)* | GTATAGATGTATGTGTTGTGTGAAGAAATAAGC | 40 |
|  | CCAATTTTCAGTAATAGGTGTGTCCC |  |
| P2 *(PDF-1.2a)* | CACACCTATTACTGAAAATTGGATGAT | 40 |
|  | TATCCCGAGGTGCATCGTTT |  |
